# Robust microorganisms for biofuel and chemical production from municipal solid waste

**DOI:** 10.1186/s12934-020-01325-0

**Published:** 2020-03-16

**Authors:** Aritha Dornau, James F. Robson, Gavin H. Thomas, Simon J. McQueen-Mason

**Affiliations:** 1 Centre for Novel Agricultural Products (CNAP), Department of Biology, University of York, Heslington, YO10 5DD York UK; 2 Department of Biology, University of York, Heslington, YO10 5DD York UK

**Keywords:** Organic municipal solid waste, MSW, Biodiesel, Bioethanol, Aviation fuel, *Rhodococcus opacus*, *Saccharomyces cerevisiae*, *Zymomonas mobilis*, Cellulosic, Biorefinery

## Abstract

**Background:**

Worldwide 3.4 billion tonnes of municipal solid waste (MSW) will be produced annually by 2050, however, current approaches to MSW management predominantly involve unsustainable practices like landfilling and incineration. The organic fraction of MSW (OMSW) typically comprises ~ 50% lignocellulose-rich material but is underexplored as a biomanufacturing feedstock due to its highly inconsistent and heterogeneous composition. This study sought to overcome the limitations associated with studying MSW-derived feedstocks by using OMSW produced from a realistic and reproducible MSW mixture on a commercial autoclave system. The resulting OMSW fibre was enzymatically hydrolysed and used to screen diverse microorganisms of biotechnological interest to identify robust species capable of fermenting this complex feedstock.

**Results:**

The autoclave pre-treated OMSW fibre contained a polysaccharide fraction comprising 38% cellulose and 4% hemicellulose. Enzymatic hydrolysate of OMSW fibre was high in d-glucose (5.5% w/v) and d-xylose (1.8%w/v) but deficient in nitrogen and phosphate. Although relatively low levels of levulinic acid (30 mM) and vanillin (2 mM) were detected and furfural and 5-hydroxymethylfurfural were absent, the hydrolysate contained an abundance of potentially toxic metals (0.6% w/v). Hydrolysate supplemented with 1% yeast extract to alleviate nutrient limitation was used in a substrate-oriented shake-flask screen with eight biotechnologically useful microorganisms (*Clostridium saccharoperbutylacetonicum*, *Escherichia coli*, *Geobacillus thermoglucosidasius*, *Pseudomonas putida*, *Rhodococcus opacus*, *Saccharomyces cerevisiae*, *Schizosaccharomyces pombe* and *Zymomonas mobilis*). Each species’ growth and productivity were characterised and three species were identified that robustly and efficiently fermented OMSW fibre hydrolysate without significant substrate inhibition: *Z. mobilis*, *S. cerevisiae* and *R. opacus*, respectively produced product to 69%, 70% and 72% of the maximum theoretical fermentation yield and could theoretically produce 136 kg and 139 kg of ethanol and 91 kg of triacylglycerol (TAG) per tonne of OMSW.

**Conclusions:**

Developing an integrated biorefinery around MSW has the potential to significantly alleviate the environmental burden of current waste management practices. Substrate-oriented screening of a representative and reproducible OMSW-derived fibre identified microorganisms intrinsically suited to growth on OMSW hydrolysates. These species are promising candidates for developing an MSW biorefining platform and provide a foundation for future studies aiming to valorise this underexplored feedstock.

## Background

The term municipal solid waste (MSW) encompasses any non-industrial waste originating from households and public or commercial institutions. Currently just over 2 billion tonnes of MSW are produced globally each year. As population growth, industrialisation and urbanisation intensify MSW volumes are projected to rise considerably to 3.4 billion tonnes per annum by 2050 [1]. Worldwide, the most common fate of MSW is to be deposited into landfill or incinerated. Both practices are unsustainable and contribute significantly to environmental pollution and climate change.

Landfills are the third largest source of anthropogenic methane emissions and are predicted to contribute significantly to global temperature rises over the next decade [2]. Even in economically developed nations, landfilling and incineration remain a primary means of MSW disposal. In the United States 52.5% of all MSW is landfilled and 12.8% is incinerated. Only about a quarter is recycled and less than 10% is composted [3]. Similarly, in the European Union only a few countries have attained recycling rates of 50% [4]. Incineration is more widespread than landfilling and usually coupled to energy generation through heat recovery [5]. Recaptured heat can be used as a domestic energy source, however, the practice still produces significant emissions in the form of carbon dioxide and nitrous oxide and requires more sophisticated infrastructure than landfilling, hampering its application in lower-income nations [6]. Innovative and holistic waste management systems are urgently needed worldwide to cope with increasing waste volumes, mitigate environmental impacts of poor waste management and enable recycling of finite resources.

MSW composition varies greatly across regions and typically consists of diverse organic and inorganic discards. In the UK 15.7 million tonnes of MSW were landfilled in 2016, of which 49% (7.7 million tonnes) was biodegradable material [7]. This organic fraction of MSW (OMSW) consists primarily of plant-derived material such as food and garden waste and pre-processed materials of plant origin such as paper and card that are rich in lignocellulose. Lignocellulose provides structure to the woody, inedible parts of plants and is comprised of up to 75% polysaccharides in the form of cellulose and hemicellulose. Lignocellulose is the most abundant renewable carbon source on the planet and the sugars that can be isolated from lignocellulosic biomass are considered the most promising sustainable alternative to petroleum in industrial manufacturing [8].

OMSW has considerable potential as a lignocellulosic feedstock as it is abundant, produced continuously and does not compete with food production. It can also be highly economical to source as landfill taxes and gate fees are often legislated to incentivise recycling and alternative routes of disposal (landfill tax rates in most European countries are at least €30 per tonne [4]). So far however research into the amenability of OMSW for producing value-added products such as biofuels has been limited compared to other feedstocks [9, 10]. Valorising OMSW for biomanufacturing poses several unique challenges, including the need for effective and commercially viable separation of the organic and inorganic fractions; inconsistent and heterogeneous feedstock composition; and the presence of metals and other pollutants in the final feedstock that could be inhibitory to enzymes and fermentative microorganisms.

OMSW composition is greatly dependent upon socioeconomic factors and prevailing local waste management practices and also varies significantly over geographic and temporal scales [1]. The complexity of OMSW contrasts starkly with agricultural and forestry by-products which generally exhibit relatively consistent compositional profiles and do not typically contain contaminants such as toxic metals [8, 11]. The abundance of organic waste in MSW ranges from 30 to 60% [1] and reports of lignocellulose content in OMSW vary between ~ 10 and 60% [10]. Typically, OMSW used for research purposes is obtained through manual sampling and sorting of MSW from local establishments [12–14] or acquired from nearby waste treatment plants [15–19]. The composition of OMSW from these sources varies significantly depending on the establishment or stage of interception and is therefore difficult to reproduce, limiting comparability between studies. Some studies have sought to improve reproducibility by using materials such as newspaper [20], food waste [21] or dog food [22] to represent OMSW. However, these substrates arguably fail to capture the heterogeneous nature of MSW-derived organic wastes.

To ensure consistency, reproducibility and real-world applicability of the OMSW used in this work, a mixture of materials was assembled to emulate the average composition of British MSW, based on statistics reported by the Department for Environment, Fisheries and Rural Affairs (DEFRA) (Additional file 1: Table S1) [23]. This constructed mixture was then processed to a biogenic fibre via a commercial autoclave processes known as the Wilson System^®^ [24], which facilitates effective separation of recyclable inorganics from organic material in mixed MSW and generates a homogeneous organic fibre with a consistent and reproducible lignocellulose fraction. Previously, a life cycle assessment (LCA) by Meng et al. [25] simulated butanol production in an MSW biorefinery based around the Wilson System^®^ autoclave and showed that a net reduction in greenhouse gas emissions of 115% could be achieved with OMSW fibre-derived liquid biofuels compared to gasoline and conventional bioethanol equivalents. Additionally, the process energy demands of the biorefinery could be fully sustained through energy recovery and biogas production.

We postulated that developing a viable bioprocess around OMSW would necessitate a highly robust and physiologically well-adapted microorganism. We therefore chose to evaluate several biotechnologically relevant microbial species for the ability to ferment hydrolysate of OMSW using a substrate-oriented screening approach. Only a handful of publications have applied a substrate-oriented approach for screening second-generation feedstocks [26–28]. Moreover, there are only few examples in the literature of microorganisms grown in monoculture on OMSW hydrolysates with the aim of producing renewable biofuels or chemicals. Published studies have reported ethanol production from autoclave pre-treated OMSW using *Saccharomyces cerevisiae* [29, 30] and *Mucor indicus* [31]; butanol production from detoxified OMSW sampled from a composting plant using *Clostridium acetobutylicum* [15, 32]; and triacylglycerol (TAG) production from OMSW obtained from a composting plant using *Cryptococcus aerius* [16].

Using a substrate-oriented approach in combination with a reproducible and realistic OMSW feedstock enables the intrinsic robustness and fermentation efficiency of industrially useful candidate species to be systematically and rigorously evaluated, thereby increasing the likelihood of developing a successful microbial platform for OMSW valorisation. Through this approach we have identified several microbial species of industrial value that demonstrated an intrinsic aptitude for growth on OMSW-derived hydrolysate. These strains are promising candidates for future studies aiming to develop a bioprocess around this underexplored feedstock.

## Results

### Composition of OMSW fibre

**Fig. 1:**
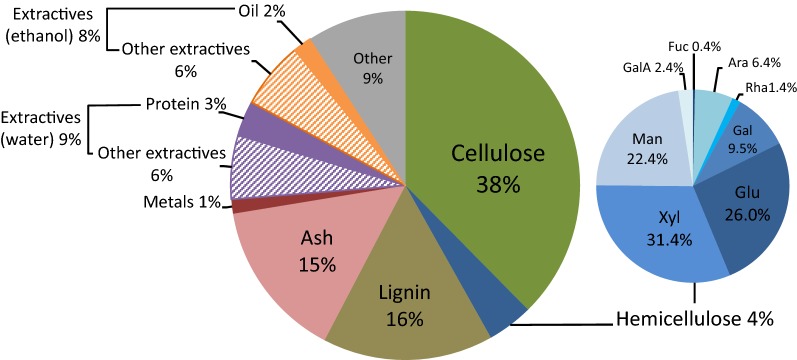
Percentage composition of OMSW fibre. A constructed batch of OMSW fibre was produced by autoclave pre-treatment of a materials mixture that reflects the composition of MSW from an average British household [23]. The dried and milled fibre was subjected to a range of compositional analyses as described in methods. Oil and protein are ethanol and water soluble, respectively, and are shown as a fraction of non-structural components extracted by water or ethanol. The monosaccharide composition of hemicellulose is shown in the smaller pie chart. All data are averages of at least triplicate analyses. Full data table given in Additional file 1: Table S1. *Glu* glucose, *Xyl* xylose, *Man* mannose, *Fuc* fucose, *Ara* arabinose, *Rha* rhamnose, *Gal* galactose, *GalA* galacturonic acid

To gain a better understanding of the final composition of the OMSW fibre the levels of relevant structural, non-structural, organic and inorganic materials were analysed by a variety of established methods. Compositional analysis accounted for 91 ± 4% of total dry mass (Fig. 1). Lignocellulose comprised approximately 58% w/w of the fibre and consisted of 65.5% cellulose (38% of total fibre), 27.6% lignin (16% of total fibre) and 6.9% hemicellulose (4% of total fibre). The major hemicellulosic sugars were d-xylose, d-glucose, d-mannose and d-galactose. The fibre also contained a large fraction of ash (15%).

Non-structural components of the biomass were extracted by Soxhlet extraction [33] with water and ethanol and made up 9% and 8% w/w dry mass, respectively. Small quantities of protein (3%) and oil (2%) were measured and accounted for as part of the extractable fractions. A wide range of common environmental metals were also detected, constituting 1% w/w dry mass of fibre in total (Additional file 1: Table S3).

The large fraction of extractable non-structural material and presence of metals highlights the highly heterogeneous nature OMSW fibre. Nevertheless, lignocellulose with a large cellulose fraction comprised over half the biomass, suggesting that OMSW fibre is a practicable fermentation feedstock.

### Analysis of OMSW fibre hydrolysate

We aimed to produce a sugar-rich hydrolysate from OMSW fibre to use in a substrate-oriented fermentation screen with a collection of biotechnologically useful microbial species. The OMSW fibre was hydrolysed in batches with the commercial enzyme cocktail Cellic Ctec3 (Novozymes) and the liquid fraction was pooled to produce a homogeneous hydrolysate. The hydrolysis yielded 75.29% of available polysaccharides (61.2% of available cellulose) with a final concentration of 78.13 ± 1.93 g/L (~ 7.8% w/v) monosaccharides (Additional file 1: Fig. S1). d-glucose, d-xylose and d-mannose were most abundant, making up ~ 98% of total sugars at 54.69 ± 1.31, 17.54 ± 1.10 and 4.25 ± 0.61 g/L, respectively. Small quantities of l-fucose, l-arabinose, l-rhamnose and d-galactose were also detected.

Marker inhibitors and common environmental metals were measured in the hydrolysate to evaluate potential toxicity to fermentative microbes and assess the degree of metal solubilisation arising through hydrolysis (Table 1). A variety of organic acids were detected, including levulinic, acetic, propionic, butyric and hexanoic acid. Levulinic and acetic acid were the most abundant, with concentrations in the mM range, while the other acids were only present at µM levels. Of the aldehyde inhibitors measured only vanillin was detected at 2.10 mM. Furfural and 5-hydroxymethylfurfural (5-HMF) were absent. A wide range of environmental metals were also found, with the majority present in the µM range.

**Table 1:** Concentration of marker inhibitors and metals detected in OMSW fibre hydrolysate and the respective minimum inhibitory concentrations (MIC) for *Escherichia coli*

Analyte*	Concentration		MIC (*E. coli*) [mM]
	[mM]	± SD	
*Acids*			
Levulinic	29.64	± 0.37	345^a^
Acetic	5.77	± 0.09	416^a^
Propionic	0.24	± 0.08	570^b^
Butyric	0.11	± 0.0027	460^b^
Hexanoic	0.11	± 0.02	12^c^
*Aldehydes*			
Vanillin	2.10	± 0.10	10^d^
5-HMF	n/d		32^d^
Furfural	n/d		36^d^
*Metals*			
Calcium	119.20	± 0.000032	n/a
Sodium	15.26	± 0.00014	n/a
Potassium	7.67	± 0.000030	n/a
Magnesium	3.65	± 0.00099	n/a
Iron	0.70	± 0.000028	1^e^
Aluminium	0.58	± 0.00014	2^f^
Zinc	0.12	± 0.000020	n/a
Manganese	0.050	± 0.000056	1^f^
Nickel	0.0061	± 0.000090	20^f^
Chromium	0.0011	± 0.00013	1^f^
Copper	0.00082	± 0.00031	1^f^
Antimony	0.00078	± 0.000053	5^f^
Vanadium	0.00072	± 0.00015	1^f^
Cobalt	0.00055	± 0.00019	1^g^
Molybdenum	0.00019	± 0.00035	n/a
Lead	0.000049	± 0.000031	5^f^

± SD, standard deviation of triplicates to 2 significant figures; n/a, not applicable; n/d, not detected

* Aldehydes and Levulinic acid were measured by ultra performance liquid chromatography with tandem mass spectrometry (UPLC-MS). Other organic acids were measured by gas chromatography with flame-ionisation detection (GC-FID). Metals were measured by ionisation coupled plasma mass spectrometry (ICPMS). All values reported to at least 2 significant figures

^a^ [34]; ^b^ [35]; ^c^ [36]; ^d^ [37]; ^e^ [38]; ^f^ [39]; ^g^ [40]

Next, we compared the concentrations of all inhibitors against minimum inhibitory concentrations (MIC) published in the literature for *E. coli* (for MICs and the associated references see Table 1). None of the inhibitors and metals measured were above the MICs for *E. coli*, although iron and aluminium were at ~ 70% and ~ 25% of inhibitory levels, respectively. Interestingly, we noticed that the concentrations of metals in the OMSW fibre hydrolysate were significantly lower than expected based on the levels of metals measured in the fibre prior to hydrolysis. We calculated the levels of metals that would theoretically be released under the hydrolysis conditions and compared these values to the actual concentrations measured in the hydrolysate. The metal content of the residual solid material left over after hydrolysis was also analysed. The results (Additional file 1: Fig. S2) demonstrated that, with the exception of potassium and sodium which are highly water soluble, the metals largely remained in the residual solid material and only about a third were solubilised into the hydrolysis liquid.

Taken together these results suggested that inhibitors were unlikely to limit fermentability of the OMSW hydrolysate as concentrations of marker inhibitory compounds were low and metals mainly remained insoluble under the hydrolysis conditions used. However, we were conscious of the fact that a range of unknown inhibitors could be present in the hydrolysate. Furthermore, not only do metal levels fluctuate on industrial scales, but metal toxicity in microbes depends greatly on pH, ion speciation and even synergistic interactions with other metals [41]. Consequently, even low levels of some metal species could become deleterious under bioprocessing conditions. We therefore decided to carry out preliminary evaluation of microbial growth on OMSW using the model fermentative microorganism *Escherichia coli*.

### Evaluating the utility of OMSW fibre hydrolysate for supporting microbial growth

**Fig. 2:**
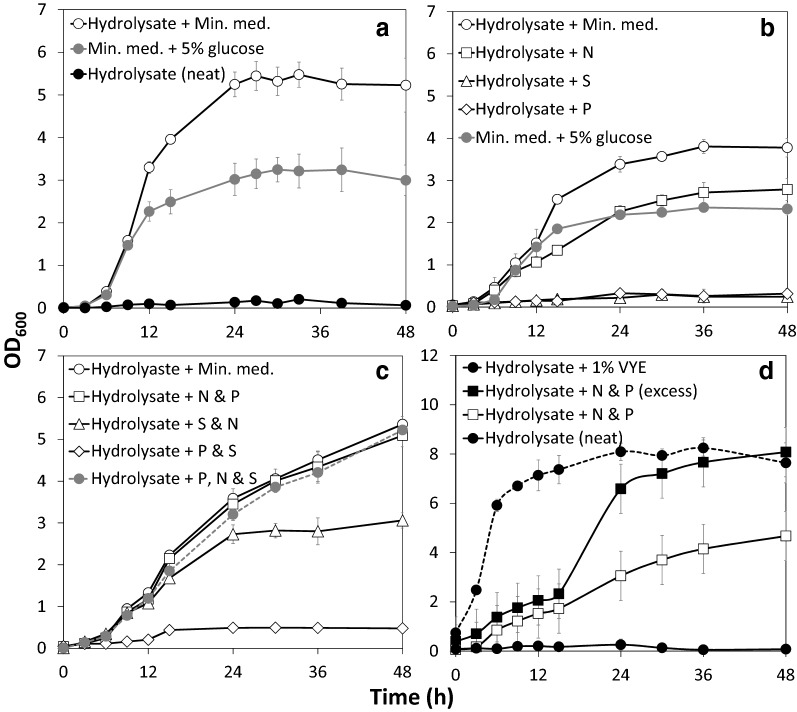
Nutrient supplementation assays with *Escherichia coli* LW06. OMSW fibre hydrolysate was supplemented with a range of nutrient sources and grown with *E. coli* LW06 over 48 h. All growth curves are averages of three biological replicates. Error bars show standard deviation from the mean. **a** Growth of *E. coli* on OMSW fibre hydrolysate supplemented with either MOPS defined medium components (‘Hydrolysate + Min. med.’) or 40 mM MOPS buffer (‘Hydrolysate (neat)’). Growth of *E. coli* on MOPS defined medium with 5% d-glucose (‘Min. med. + 5% glucose’) shown as positive control. **b** Growth of *E. coli* on OMSW fibre hydrolysate supplemented with either 0.3 mM K_2_SO_4_ (‘Hydrolysate + S’), 10 mM NH_4_Cl (‘Hydrolysate + N’) or 0.5 mM K_2_HPO_4_ (‘Hydrolysate + P’) (concentrations the same as MOPS defined medium). ‘Hydrolysate + Min. med.’ and ‘Min. med. + 5% glucose’ as in **a** (growth assays repeated in parallel for comparison). **c** Growth of *E. coli* on OMSW fibre hydrolysate supplemented with either 10 mM NH_4_Cl and 0.5 mM K_2_HPO_4_ (‘Hydrolysate + N & P), 0.5 mM K_2_HPO_4_ and 0.3 mM K_2_SO_4_ (‘Hydrolysate + P & S), or 0.5 mM K_2_HPO_4_, 10 mM NH_4_Cl and 0.3 mM K_2_SO_4_ (‘Hydrolysate + P, N & S’). Concentrations used are the same as for MOPS defined medium. ‘Hydrolysate + Min. med.’ as in **a** (growth assay repeated in parallel for comparison). **d** Growth of *E. coli* on OMSW fibre hydrolysate supplemented with 1% vitamin-enriched yeast extract (‘Hydrolysate + 1% VYE’) or an excess of ammonium and phosphate (20 mM NH_4_Cl and 1 mM K_2_HPO_4_) (‘Hydrolysate + N & P (excess)’). ‘Hydrolysate + N & P’ as in **c** and ‘Hydrolysate (neat)’ as in **a** (growth assays repeated in parallel for comparison)

Preliminary attempts to culture the ethanol producing *Escherichia coli* strain LW06 solely on OMSW hydrolysate under aerobic conditions were unsuccessful. We therefore carried out a series of assays with *E. coli* LW06 to determine the cause of growth limitation (Fig. 2).

When OMSW hydrolysate was supplemented with all chemical components necessary for growth at the same concentration as MOPS defined medium, (a minimal medium for Enterobacteria developed by Neidhardt et al. [42]), *E. coli* was able to grow to an OD_600_ of ~ 5.5 over 30 h. By comparison, cells grown on the control medium (MOPS defined medium with 5% w/v glucose) produced about 40% less biomass (Fig. 2a). This indicated that nutrient restriction was the primary cause of growth limitation rather than substrate inhibition. Lignocellulosic hydrolysates are often low in nitrogen and phosphorus compared to first generation feedstocks and may require nutrient supplementation to be viable for fermentation [43]. To determine specifically which nutrients were limiting, the hydrolysate was supplemented with a source of sulphate (K_2_SO_4_), ammonium (NH_4_Cl) and phosphate (K_2_HPO_4_) at the same concentrations used in MOPS defined medium.

Addition of either phosphate or sulphate alone did not significantly increase growth. Ammonium supplementation produced growth to levels comparable with the control medium but could not restore growth to the levels observed on OMSW fibre hydrolysate supplemented with defined medium (Fig. 2b). This indicated that a second nutrient was limiting further growth beyond nitrogen. The hydrolysate was therefore supplemented combinatorially with ammonium, phosphate and sulphate (Fig. 2c). Hydrolysate supplemented with ammonium and phosphate produced growth equivalent to hydrolysate supplemented with defined medium and hydrolysate supplemented with ammonium, phosphate and sulphate. Taken together these results demonstrated that growth of *E. coli* LW06 on the hydrolysate was limited by a significant deficiency in nitrogen and further limitation in phosphate.

Slight differences in growth rate and time of entry into stationary phase were observed between experiments (Fig. 2a–c). This variation is likely due to differences in seed culture growth stage at inoculation because growth trends are internally consistent within each experiment (i.e. growth on Hydrolsyate + Min. med. is greater by an OD_600_ of ~ 1 in Fig. 2A compared to Fig. 2B, but this is also the case for Min. med. + 5% glucose). Nevertheless, these growth assays showed that nutrient supplemented OMSW hydrolysate supports excellent growth of *E. coli* without notable inhibition and suggests that the hydrolysate is likely to be tolerated by other fermentative microbes.

In industry nutritional fermentation supplements are usually derived from the low-cost abundant waste products of other industries, for example corn steep liquor, yeast autolysate or casein hydrolysate [44]. When *E. coli* LW06 was grown on OMSW fibre hydrolysate supplemented with 1% Vitamin-enriched yeast extract (VYE) (a substitute for yeast autolysate) growth improved significantly (Fig. 2d, ‘Hydrolysate + 1% VYE’). The cells entered exponential phase more rapidly and reached a final OD_600_ of ~ 8.0, almost twice the biomass achieved on hydrolysate supplemented with phosphate and ammonium (Fig. 2d, ‘Hydrolysate + N and P’). This level of growth could be recapitulated when cells were grown on hydrolysate supplemented with excess ammonium (20 mM NH_4_Cl_2_) and phosphate (1 mM K_2_HPO_4_), although the initial lag phase was extended under these conditions (Fig. 2d, ‘Hydrolysate + N and P (excess)’). We therefore decided that supplementing the OMSW fibre hydrolysate with 1% VYE would be the most industrially pertinent method of providing accessible nitrogen and phosphate when assaying fermentability with our diverse collection of microorganisms.

### Characterising growth of eight microbial species on OMSW fibre hydrolysate

**Fig. 3:**
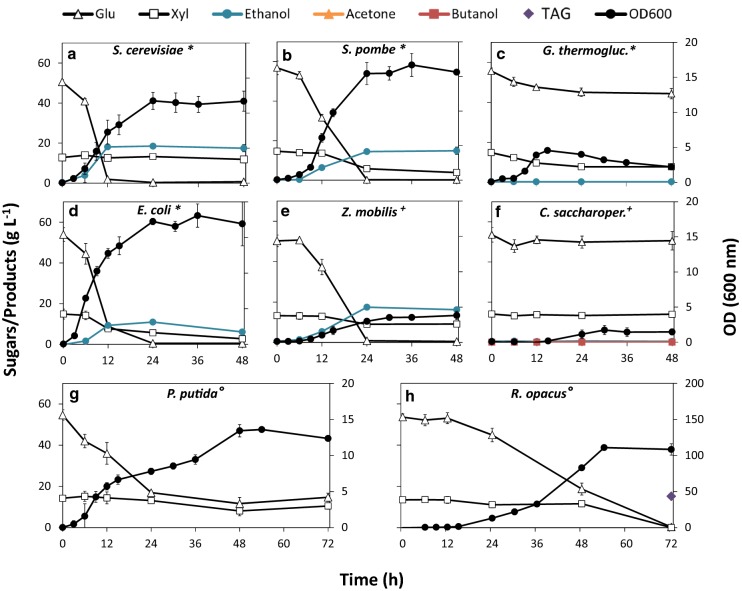
Fermentation kinetics of eight different microorganisms grown on OMSW fibre hydrolysate. Each species (*Saccharomyces cerevisiae*, *Schizosaccharomyces pombe*, *Geobacillus thermoglucosidasius*, *Clostridium saccharoperbutylacetonicum*, *Escherichia coli*, *Zymomonas mobilis*, *Pseudomonas putida* and *Rhodococcus opacus*) was grown separately in triplicate on 10 mL OMSW fibre hydrolysate supplemented with 1% vitamin-enriched yeast extract and buffered with 40 mM MOPS. Accumulation of products (ethanol, butanol, acetone or triacylglycerol (TAG)) and consumption of glucose (triangle) and xylose (square) are plotted on the primary Y-axis. Optical density at 600 nm (secondary Y axis) was used as a measure of biomass production. All species were inoculated to a starting OD_600_ of 0.05. Note that TAG yield was only measured at 72 h. Culture conditions: *Semi-Aerobic in shake flasks with airlocks; ^+^Anaerobic in serum bottles; circles Aerobic in shake flasks

To evaluate the potential of OMSW fibre as a feedstock for biofuel and chemical production we selected diverse microorganisms of industrial interest and characterised their ability to ferment hydrolysate of OMSW fibre. We selected the microorganisms based on one or more of the following: biotechnological utility; genetic tractability; biofuel or chemical production; and inhibitor tolerance. Each species was inoculated into 10 mL OMSW fibre hydrolysate supplemented with 1% VYE and incubated under optimal growth conditions (Table 4). Samples were taken at regular intervals for up to 72 h and used to measure optical density at 600 nm (OD_600_), sugar utilisation and product accumulation. All species grew on the OMSW fibre hydrolysate. However, the dynamics of growth, carbon consumption and product synthesis were unique to each. The time course kinetics of these variables is shown in Fig. 3, providing an overview of the fermentation dynamics for each species. To quantitatively compare the relative performance of the different microbes, key yield parameters were calculated for each fermentation (Table 2).

**Table 2:** Key fermentation yield parameters for eight species grown on OMSW fibre hydrolysate

Species	Strain	Product	Time (h)	Glucose used (%)	Xylose used (%)	Total sugars (used)^a^	Mean ± SD					Yield per tonne (kg/t)^e^	Theoretical max. yield (kg/t)^f^
							Final CDW (g/L)	Product titre (g/L)	Productivity (g/L/h)^b^	P/S (g/g)^c^	Percentage yield (%)^d^		
*P. putida*	NCIMB8249	None	72	73.1	n/a	63.4	2.5 ± 0.4	n/a	n/a	n/a	n/a	n/a	n/a
*C. saccharoperbutylacetonicum*	DSM14923	Butanol	48	5.6	0.0	4.4	0.7 ± 0.1	n/d	n/a	n/a	n/a	n/a	n/a
*G. thermoglucosidasius*	DSM2542	Ethanol	48	20.3	47.5	26.0	0.4 ± 0.1	n/d	n/a	n/a	n/a	n/a	n/a
*E. coli*	LW06	Ethanol	48	99.1	61.6	91.0	9.0 ± 0.8	10.9 ± 0.5	0.46 ± 0.02	0.17 ± 0.01	34 ± 2	55	70
*S. pombe*	JB953	Ethanol	48	99.8	n/a	99.8	3.3 ± 0.1	14.9 ± 1.9	0.31 ± 0.04	0.26 ± 0.03	51 ± 7	74	101
*Z. mobilis*	DSM424	Ethanol	24	98.4	n/a	98.4	1.3 ± 0.2	17.5 ± 0.3	0.73 ± 0.01	0.35 ± 0.01	69 ± 1	87	136
*S. cerevisiae*	ATCC200062	Ethanol	24	99.3	n/a	99.3	2.5 ± 0.1	18.1 ± 1.3	0.75 ± 0.06	0.36 ± 0.03	70 ± 5	90	139
*R. opacus*	MITXM-61	TAG	72	99.5	100.0	99.6	32.7 ± 0.4	15.2 ± 1.1	0.21 ± 0.02	0.23 ± 0.02	72 ± 5	76	91

TAG, Triacylglycerol; n/a, not applicable; n/d, not detected; ± SD, standard deviation of triplicate measurements

The equations used to calculate these variables are given in Additional file 1: Additional Methods

^a^Percentage of metabolically available sugars consumed based on initial d-glucose and d-xylose concentrations

^b^Grams of product produced per litre of fermentation medium per hour, also known as process productivity, (g/L/h)

^c^Product to substrate yield ratio (grams of product per gram of sugar fermented)

^d^Product titre attained by fermentation, given as a percentage of the theoretical maximum fermentation yield from sugars. Assuming theoretical maxima of 0.316 g/g sugar for triacylglycerol and 0.511 g/g for ethanol. Note that these figures may be overestimates as only the major carbon sources d-glucose and d-xylose were accounted for. Less abundant sugars or unaccounted carbon sources could have contributed to the yield

^e^kg of product that could be produced from one tonne of OMSW fibre, based on observed conversion efficiencies

^f^kg of product that could be produced from one tonne of OMSW fibre, assuming complete conversion of sugars in hydrolysis

The fermentation kinetics in conjunction with the calculated yield parameters enabled each species’ relative performance on the OMSW fibre hydrolysate to be compared. The poorest performing strains consumed less than 50% of metabolically available sugars and did not synthesise the desired fermentation product; this included *Geobacillus thermoglucosidasius* DSM2542 and *Clostridium saccharoperbutylacetonicum* DSM14923. *G. thermoglucosidasius* grew relatively rapidly over the first 15 h, metabolising d-glucose and d-xylose simultaneously (Fig. 3c) [45, 46]. Growth then abruptly ceased, although 74% of d-glucose and d-xylose remained. No ethanol was detected in the medium, indicating growth was not constrained by product inhibition but likely related to substrate inhibition. *C. saccharoperbutylacetonicum* exhibited an extended lag phase for ~ 12 h and only grew slowly to an OD_600_ of 1.72 ± *0.65* over 24 h before growth stopped (Fig. 3f). No acetone, butanol or ethanol were detected in the medium at any time point and final pH was 4.5, ruling out the possibility of autoacidification and indicating that growth was limited by intolerance to an unknown component of the hydrolysate.

Species that performed moderately well include *Pseudomonas putida* NCIMB8249, *E. coli* LW06 and *Schizosaccharomyces pombe* JB953. *P. putida* was chosen for its inhibitor tolerance as feedstock toxicity was initially unknown. Although *P. putida* reached a relatively high biomass concentration (2.5 g/L), only 73.1% of d-glucose was utilised (Table 2). The overall growth trend was biphasic, with a brief lag phase after 12 h, followed by growth recommencing after 36 h. The final stationary phase was reached at 48 h, coinciding with the cessation of glucose metabolism (Fig. 3g). The final pH of the fermentation medium was ~ 3.0 in all three replicates. A major limitation for the industrial application of *P. putida* is that it does not possess all acid stress response pathways typically found in Enterobacteria [47]. It is therefore likely that growth was inhibited through autoacidification. This issue could be circumvented by using a stronger buffer or in-line pH control during the fermentation.

*Escherichia coli* LW06 can be induced with IPTG to produce ethanol through the Entner-Doudoroff pathway [48]. Cells induced with 1 mM IPTG produced 10.9 g/L ethanol (34% of theoretical yield) and used 91% of available d-glucose and d-xylose (Table 2). Carbon catabolite repression was observed, with d-glucose being used preferentially over d-xylose. Overall about three-fold more carbon was dedicated to biomass production than ethanol synthesis, possibly due to the heterologous nature of the ethanol pathway in this strain. Assuming complete conversion of polysaccharides in hydrolysis, *E. coli* LW06 could produce 70 kg of ethanol from one tonne of OMSW fibre.

The fission yeast *S. pombe* is a model organism in molecular genetics studies [49], but has been highlighted as a promising industrial bioethanol producer [50–52]. *S. pombe* demonstrated robust growth on the OMSW hydrolysate and utilised all available glucose within 24 h. However, ethanol was only produced to 51% of theoretical fermentation yield, equivalent to 14.9 ± 1.9 g/L. This species is unable to ferment d-xylose, but many yeasts are able to assimilate d-xylose and synthesise xylitol [53], which may account for the decline of d-xylose in the fermentation (Fig. 3b). *S. pombe* did not show any obvious signs of product inhibition but only produced half the theoretically possible ethanol titre, indicating that yields could be improved further by optimising fermentation conditions. Overall, based on the observed fermentation productivity, *S. pombe* could produce 87 kg of Ethanol per tonne of OMSW. Theoretically this titre could rise to 101 kg/t if hydrolysis was optimised (Table 2).

The most promising strains identified were *Saccharomyces cerevisiae* ATCC200062, *Zymomonas mobilis* DSM424 and *Rhodococcus opacus* MITXM-61. The ethanol producing species *S. cerevisiae* (Fig. 3a) and *Z. mobilis* (Fig. 3e) achieved near maximum theoretical yields within 24 h (Table 2). Yields were comparable when accounting for the standard deviation, with *S. cerevisiae* producing 18.1 ± 1.3 g/L ethanol (70 ± 5% of theoretical yield) and *Z. mobilis* producing 17.5 ± 0.3 g/L ethanol (69 ± 1% of theoretical yield) with productivities of 0.73 ± 0.01 g/L/h and 0.75 ± 0.06 g/L/h, respectively. Notably, total biomass production was 46% lower in *Z. mobilis* compared to *S. cerevisiae* despite attaining near identical ethanol yields. The high specific productivity of *Z. mobilis* compared to yeast is well established [54] and further supported by our results.

Based on their performance in the screen, a bioprocess with *S. cerevisiae* and *Z. mobilis* could produce 87 kg and 90 kg of ethanol, respectively, from one tonne of OMSW. Assuming complete hydrolysis of all polysaccharides in the OMSW fibre, fermentation yields could theoretically rise to 136 and 139 kg/t (Table 2). Despite their impressive performance, the inability of *S. cerevisiae* and *Z. mobilis* to metabolise D–xylose limits their productivity on OMSW hydrolysate. Metabolic engineering of pentose fermentation is an area of significant research [55] and promising d-xylose utilising strains have already been developed for both species [56, 57]. The *S. cerevisiae* strain used in this project, ATCC200062 (also NREL D5A), is genetically derived from Red Star^®^ baker’s yeast and was selected because it has repeatedly been shown to robustly ferment lignocellulosic feedstocks [58, 59]. Interestingly, a recent study [60] demonstrated that ATCC200062 can be evolutionarily engineered to ferment xylose. Employing engineered xylose utilising strains could further improve the productivity of these robust species on OMSW fibre hydrolysate.

*Rhodococcus opacus* is an oleaginous bacterium that produces intracellular stores of triacylglycerol (TAG). TAG is a promising precursor for the production of biodiesel and aviation fuel, but can also be derivatised to produce a range of valuable chemicals including polymers and surfactants [61–63]. *R. opacus* MITXM-61 was engineered by Kurosawa et al. [64] for simultaneous utilisation of d-glucose and d-xylose in lignocellulosic hydrolysates. In this study *R. opacus* MITXM-61 was the top performing strain in terms of sugar utilisation. All available glucose and xylose were consumed in parallel within 72 h and the culture reached a high OD_600_ of ~ 110, equating to a final cell dry weight of 32.7 g/L.

Our analysis showed that TAGs were accumulated to 48.9% of cell dry weight after 72 h, which was the time point when all glucose and xylose was depleted and thus cells were most likely to contain the greatest TAG titre [65]. Only an end-point measure was taken due to the need for at least 5 mg dry cell material for TAG quantification (see Additional file 1: Additional Mathods). A time course would have required removal of large volumes of the fermentation medium which could have perturbed fermentation dynamics. We calculated that the measured TAG yield was equivalent to 72% of the maximum theoretical titre. Upon cell lysis this would produce about 15.2 ± 1.1 g/L TAG-derived FAs. Based on these results, we calculated that about 76 kg of TAG could be produced per tonne of OMSW fibre, rising to 91 kg/t if feedstock conversion is optimised (Table 2).

The TAG produced by *R. opacus* grown on OMSW fibre had a FA profile typical of this species, comprising mostly C:14–C:18 FAs with an abundance of Palmitic acid (C16:0) (Table 3) [66]. To evaluate the viability of TAG-derived FAs from *R. opacus* for biodiesel production the Cetane number (CN) was calculated using equations developed by Klopfenstein [67]. CN is a dimensionless number used to measure the combustion and ignition potential of a biodiesel relative to Cetane (n-hexadecane), a highly ignitable straight chain hydrocarbon [68]. EU specifications stipulate a minimum CN of 51 for biodiesel, with a minimum Cetane index (CN_i_) of 46 for all constituent fatty acid methyl esters (FAMES) [69]. Only eight of the thirty FAs had a CN_i_ below the threshold of 46 and these only made 1.67% of the total FA profile (Table 3). The total mixture of FAMEs isolated from *R. opacus* had a CN of 62.5, indicating that TAGs from *R. opacus* grown on OMSW fibre could theoretically be converted directly to high-quality biodiesel, on par with oil-crop derived biodiesels currently produced from jatropha and palm [70].

**Table 3:** Fatty acid composition profile of *Rhodococcus opacus* MITXM-61 and the calculated Cetane index of each fatty acid

FA	C:D	%	± SD	CN_i_
Capric	C10:0	0.04	± 0.02	60.9
Undecylic	C11:0	0.12	± 0.00	62.3
Lauric	C12:0	0.14	± 0.02	63.7
Tridecylic	C13:0	0.03	± 0.00	65.1
Myristic	C14:0	2.17	± 0.16	66.5
Myristoleic	C14:1 [9]	0.03	± 0.00	50.6
Pentadecanoic	C15:0	5.96	± 0.41	67.9
*Cis*-10-pentadecenoic	C15:1 [5]	0.45	± 0.03	52.0
Palmitic	C16:0	28.84	± 1.96	69.3
Hypogeic	C16:1 [7]	9.06	± 0.72	53.4
Heptadecanoic	C17:0	10.88	± 0.78	70.7
*Cis*-10-heptadecenoic	C17:1 [10]	13.65	± 0.96	54.8
Stearic	C18:0	5.28	± 0.36	72.1
*Trans*-9-octadecenoic	C18:1 [9]	1.93	± 0.18	56.2
*Cis*-9-octadecenoic	C18:1 [9]	18.65	± 1.49	56.2
*Cis*-11-octadecenoic	C18:1 [11]	0.45	± 0.09	56.2
9-*trans*, 12-*trans*-octadecadienoic	C18:2 [9, 12]	0.02	± 0.02	40.3
9-*cis*, 12-*cis*-octadecadienoic	C18:2 [9, 12]	0.07	± 0.01	40.3
y-Linoleic	C18:3 [6, 9, 12]	1.18	± 0.09	24.4
Stearidonic	C18:4 [6, 9, 12, 15]	0.03	± 0.00	8.5
Arachidic	C20:0	0.23	± 0.02	74.9
Gondoic	C20:1 [11]	0.06	± 0.00	59.0
*Cis*-13-eicosenoic	C20:1 [13]	0.04	± 0.04	59.0
Homo-y-linolenic	C20:3 [8, 11, 14]	0.06	± 0.03	27.2
Arachidonic	C20:4 [5, 8, 11, 14]	0.04	± 0.02	11.3
Eicosapentaenoic	C20:5 [5, 8, 11, 14, 17]	0.07	± 0.01	− 4.6
Behenic	C22:0	0.14	± 0.03	77.7
Erucic	C22:1 [13]	0.10	± 0.02	61.8
Docosadienoic	C22:2 [13, 16]	0.21	± 0.02	45.9
Nervonic	C24:1 [15]	0.07	± 0.01	64.6
FA (% total)		100.00	± 2.91	
FA (% of cell dry weight)		48.91	± 1.42	
CN (total)				62.5

Yields are given as the percentage (w/w) of total fatty acids (FA) with standard deviation (± SD) of triplicate measurements. Common names are given where available

C:D, lipid number, expressed as the number of carbon atoms to double bonds. Double bond locations are numbered in parentheses. CN_i_, cetane index, measures the combustibility and ignitability of individual FAMEs. CN, cetane number, measures the combustibility and ignitability of biodiesel mixture

## Discussion

### OMSW fibre as a feedstock for bio-manufacturing

The organic fraction of MSW has potential as an abundant renewable feedstock for sustainable production of fuels and bio-based chemicals. However, investigations into its utility are limited by the challenging technical obstacle of finding a consistent material that accurately mimics the complex, heterogeneous nature of OMSW. The constructed mixture of organic MSW fibre presented in this study aims to overcome these limitations. The Wilson System^®^ is a commercially viable autoclave process that allows for reproducible production of a homogeneous lignocellulose-rich feedstock from a controlled mixture of MSW. To ensure our feedstock would be replicable, consistent and representative of real-world OMSW fibre, we based the composition of our MSW on averages for MSW generated in the United Kingdom published by the Department of Environment, Food and Rural Affairs [23]. Results from this study are therefore also pertinent to other nations with similar consumption patterns.

Autoclaving is an established industrial-scale process that is widely employed in the waste industry to rapidly, hygienically and effectively recover resources from MSW [71, 72]. Compositional profiles for autoclave pre-treated OMSW have been reported in three other studies [10, 29, 30]. All, including this study, report about 50% greater polysaccharide levels than the averages calculated by Barampouti et al. [10] in their review of the relevant literature. This indicates that autoclaving is also an effective strategy for isolating and concentrating organic materials in OMSW. In general, autoclaving overcomes the inherent challenges associated with isolating OMSW from a complex and heterogeneous MSW mixture while also acting as a mild pre-treatment.

There is also a general consensus in the literature that hydrothermal pre-treatments like autoclaving produce fewer inhibitors than other processes but can effectively increase cellulose accessibility in a variety of feedstocks [73]. Hydrolysis methodology was not a major focus of this work as it has been explored in several other studies [17, 29, 74–76]. However, we showed that the OMSW fibre produced through autoclave pre-treatment of mixed MSW could be directly hydrolysed with the enzyme cocktail Cellic Ctec3 (Novozymes) without the need for further pre-treatment. Hydrolysis yield was 75% of total polysaccharides but this was achieved using a relatively high enzyme loading. Yields and efficiency could undoubtedly be improved by using a dedicated hydrolysis vessel with mixing capabilities. Exploration of different pre-treatment methods, such as alkali, dilute-acid or steam explosion could also help improve sugar accessibility [77].

Due to its heterogeneous nature and compositional variability, OMSW is likely to contain a diversity of chemicals that are uncommon in agriculturally derived feedstocks. Two studies by Farmanbordar et al. [15, 32] reported total phenolics and tannins in OMSW pre-treated with dilute acid and organosolv, while Ghanavati et al. [16] reported 5-HMF and furfural concentrations in detoxified OMSW hydrolysate. To our knowledge this study presents the most comprehensive analysis of key lignocellulose-derived inhibitors in an OMSW-derived hydrolysate published to date. Based on our analysis of the hydrolysate produced in this study, levels of inhibitory compounds were below the threshold of toxicological concern for *E. coli* (Table 1). Furfural and 5-HMF, two furaldehyde inhibitors that pose a significant problem in pre-treated lignocellulosic feedstocks [78], were not detected in the OMSW fibre hydrolysate. However, the presence of low concentrations of levulinic acid indicates that some 5-HMF was originally present but then degraded [73]. Unfortunately, formic acid could not be measured to determine if furfural was similarly degraded but very little would be expected considering the low levels of hemicellulose in the fibre. Overall, these results demonstrate that the autoclave process is highly advantageous for pre-processing OMSW—not only is it an efficient method for homogenising and isolating OMSW from mixed MSW but it also acts as an effective pre-treatment that minimises inhibitor formation. This is a notable advantage in a feedstock that is already inherently complex and contaminated with unknown compounds and metals.

The presence of metals is a limitation that is unique to OMSW-derived feedstocks. Many of the metal species found in this study can be significantly inhibitory to microorganisms at high concentrations and under some ionisation states and pH conditions. However, we demonstrated that hydrolysis performed with MSW fibre acidified to pH 5 with H_2_SO_4_ produces a hydrolysate in which the majority of metals remain insoluble (Additional file 1: Fig. S2). Although the OMSW hydrolysate was largely tolerated by the microorganisms’ trialled in this study, the composition of real-world OMSW is highly variable and metal levels may fluctuate between batches in an industrial context. Farmanbordar et al. [15] demonstrated that over-liming can also be used for metal detoxification of OMSW prior to hydrolysis, presenting an additional option to mitigate metal toxicity if required. Further work is necessary to better evaluate the effects of variable MSW-derived metal levels on fermentation efficiency.

### Vitamin-enriched yeast extract as a nutrient supplement

We demonstrated that MSW fibre hydrolysate is greatly limited in microbially accessible nitrogen and, to a lesser extent, phosphate (Fig. 2). After our initial nutrient supplementation assays with NH_4_Cl and K_2_HPO_4_ we chose to supplement 1% vitamin-enriched yeast extract (VYE) as a nutrient source for all subsequent fermentations. On an industrial scale VYE could be substituted with autolysed spent yeast which is high in vitamins, nitrogen and phosphate and is generated in substantial volumes in the plethora of breweries operating across the UK. A medium sized brewery (> 1000–2000 L batch capacity) may produce thousands of kg of spent yeast per week, of which 40–70% is disposed into local sewage works as demand for alternative applications (animal feed, anaerobic digestion or fertiliser) is limited and off-site transportation expensive [79]. The potential for combining two waste streams, OMSW and spent yeast, is an appealing concept for a sustainable biorefinery.

### Promising species for industrial production of fuels and chemicals from OMSW fibre

Few microbial species have been grown in monoculture on OMSW-derived sugars with the aim of producing biofuels or chemicals [15, 16, 29–31]. To our knowledge, this is the first time growth on hydrolysate of OMSW has been demonstrated for *C. saccharoperbutylacetonicum*, *E. coli*, *G. thermoglucosidasius*, *P. putida*, *R. opacus*, *S. pombe* and *Z. mobilis*. All species evaluated in this study grew on the OMSW fibre hydrolysate but their relative productivities varied significantly (Fig. 3).

The poorest performing strains (*C. saccharoperbutylacetonicum* and *G. thermoglucosidasius*) were easily identified as they used less than 50% of metabolically available sugars and entered stationary phase prematurely, indicative of substrate inhibition. These species are therefore less desirable candidates for use in an OMSW fibre-based bioprocess. Previous work has shown that Clostridia grown on lignocellulosic hydrolysates are primarily inhibited by phenol, furfural and formic acid [80]. Furfural was absent and we were unable to measure formic acid, but 21 mM vanillin, the marker inhibitor for phenolics, was detected (Table 2). Furthermore, the OMSW fibre hydrolysate was dark brown in colour, which is indicative of a high concentration of lignin-derived polyphenolic compounds.

Phenolic compounds such as tannins can incapacitate enzymes through hydrogen crosslinking with carbonyl groups [81]. Farmanbordar et al. [15] found that tannins present in OMSW hydrolysate greatly inhibited butanol production in *Clostridium acetobutylicum*. Thus lignin-derived phenolic and polyphenolic compounds may be responsible for the poor growth observed in the closely related *C. saccharoperbutylacetonicum*. Extracting tannins from the OMSW prior to hydrolysis could alleviate inhibition in *C. acetobutylicum* [15], however, on industrial scales this would require additional processing steps that may be uneconomical. Another option would be to engineer phenol tolerance using the ever increasing repertoire or genetic and synthetic biology tools available for *Clostridia* [82].

Gram-positive species are typically more susceptible to phenol inhibition, possibly due to the greater protection from hydrogen bonding afforded by the Gram-negative outer membrane [81]. *G. thermoglucosidasius* is Gram-positive and may have been more susceptible to inhibition by phenolics. On the other hand, *R. opacus*, the other Gram-positive species in our collection, did not show any obvious signs of substrate inhibition (Fig. 3h), likely because it has an unusually complex mycolic-acid envelope which has been associated with phenol tolerance and even enables growth on phenol as a sole carbon source [83].

Species that emerged as the most promising candidates for OMSW fermentation include *S. cerevisiae*, *Z. mobilis* and *R. opacus*. All three species depleted metabolically available sugars and attained product titres close to theoretical maximum. *S. cerevisiae* and *Z. mobilis* were closely tied in terms of productivity (0.73 ± 0.01 g/L/h and 0.75 ± 0.06 g/L/h, respectively) (Table 2). Both species are long established in the literature as outstanding candidates for ethanol production [84, 85] and their intrinsic aptitude for fermenting a wide array of lignocellulosic feedstocks is confirmed further by their efficient and robust performance on OMSW fibre hydrolysate.

Interestingly, ethanol production by *S. cerevisiae* plateaued after 12 h, with only a marginal, statistically insignificant rise in ethanol titre between 12 and 24 h (18.0 ± 1.0 g/L to 18.1 ± 1.3 g/L) (Fig. 3a). Within the same timeframe OD_600_ continued to increase significantly from 7.29 ± 1.70 to 11.75 ± 1.23. This indicates that with some minor optimisation of fermentation conditions maximal ethanol production could be achieved within 12 h. This would increase productivity to an estimated 1.5 g/L/h, which is above the minimum viable productivity for bioethanol producing strains, calculated to be > 1 g/L/h by Dien et al. [86].

*Rhodococcus opacus* produced TAG from OMSW hydrolysate to 48.91% of CDW (72% of theoretical fermentation yield on glucose). Fatty acid yields vary by carbon source and the maximum reported in the literature for this species is 76% of CDW from gluconate [66, 87]. The TAG titre achieved with *R. opacus* was 15.2 ± 1.1 g/L, slightly lower by mass than the ethanol titres of *S. cerevisiae* and *Z. mobilis*, however, TAG biosynthesis differs metabolically and physiologically from ethanol fermentation and is also economically distinct as it competes primarily with palm oil for biodiesel production. Making direct comparisons between ethanologenic and oleaginous species is therefore challenging. However, *R. opacus* was arguably the most productive of the three species as it was able to access a greater fraction of the total available carbon by efficiently and concurrently fermenting d-glucose and d-xylose. Based on the hydrolysis efficiencies attained in this study it was calculated that approximately 94 g of TAG could be produced per kg of OMSW fibre. This strain has been shown to be highly productive even under glucose concentrations of 300 g/L under batch fermentation conditions [88] and we calculated that increasing hydrolysis efficiency could theoretically increase yields up to 91 kg/t.

Overall, the total TAG titre attained on OMSW fibre (15.2 ± 1.1 g/L) corresponds well with work by Kurosawa et al. [64] wherein MITXM-61 grown on corn stover hydrolysate produced a 15.9 g/L TAG. However, the overall productivity of MITXM-61 was significantly greater on OMSW fibre (0.21 ± 0.02 g/L/h) compared to corn stover (0.13 g/L/h) [64] due to a shorter lag phase. There was a 48 h lag phase before growth commenced on corn stover [64], whereas in the two fermentation trial carried out with *R. opacus* on OMSW fibre, the lag phase only lasted ~ 12 h (Fig. 2-H). Similarly, there was a ~ 96 h lag phase during growth of *R. opacus* on hardwood pulp [89]. This demonstrates that OMSW fibre hydrolysate may be a more favourable feedstock for *R. opacus* compared to other lignocellulosic hydrolysates. A critical parameter for attaining high TAG yields is the carbon to nitrogen ratio [88] and further optimisation of nutrient supplementation and fermentation conditions such as aeration and pH could potentially reduce lag times further and increase growth rate.

The identity and abundance of major FAs in *R. opacus* grown on OMSW fibre hydrolysate was consistent with those reported in previous studies of MITXM-61 grown on a mixture of glucose and xylose, and on alkali pre-treated corn stover [64, 90]. Furthermore, FAs extracted from *R. opacus* grown on OMSW fibre had a calculated CN of 62.5. Comparably, Fei et al. [90] reported a CN of 60 for *R. opacus* MITXM-61 grown on glucose and xylose. These results demonstrated that the FA profile of *R. opacus* was not significantly perturbed by the complex and heterogeneous composition of OMSW fibre hydrolysate and therefore TAG produced by *R. opacus* grown on OMSW fibre hydrolysate could be used directly for biodiesel production. This also presents a potential route for producing renewable aviation biofuels from OMSW, as a variety of established hydrocracking and hydroisomerization technologies exist for converting TAG into aviation-grade paraffins [70].

Overall, the high-performing species identified in this study have considerable promise for production of renewable biofuels and chemicals from OMSW, but further work is needed to assess viability for industrial-scale fermentations. In particular, evaluation of growth in bioreactors is critical for determining bioprocess scalability [91, 92] and life cycle assessment (LCA) should be used to holistically compare the performance of different microbial platforms [25, 93]. Downstream processing methods are also a major factor to consider when assessing feasibility for industrial applications [94]. A key limitation of microbial lipid bioprocesses is the cost of isolating product from cells [95] and practical and sustainable methods for commercial-scale extraction of microbial lipids are needed to advance sustainable production.

Lastly, species that showed moderate performance on OMSW hydrolysate should not be overlooked. *S. pombe* produced a high titre of ethanol (14.9 ± 1.9 g/L) but diverted more carbon to biomass production than its close relative, *S. cerevisiae* (3.35 ± 0.10 g/L and 2.50 ± 0.10 g/L, respectively). *S. pombe* is nevertheless an interesting species that has not been studied in great detail for bioethanol production despite sharing many desirable traits with *S. cerevisiae*, including flocculability, genetic tractability, and tolerance to ethanol and osmotic stress [50]. Wider exploration of strains in this species may identify useful industrial fermentation phenotypes, as has already been done for wine making [52]. Similarly, *E. coli* grew robustly on the hydrolysate and produced ethanol to 34% of theoretical yield. As *E. coli* is a well-established host for genetic engineering this opens up the potential for producing a broad range of natural products, fuels and chemicals from OMSW [96–98]. *P. putida* achieved relatively high biomass density on the OMSW fibre hydrolysate despite not consuming all metabolically available sugars. Nikel and de Lorenzo [99] have demonstrated that *P. putida* can be engineered to produce ethanol and exhibits superior tolerance to ethanol stress when compared to *E. coli*. It has also been shown to produce polyhydroxyalkanoates, which can be used as monomers for biopolymer production [100]. These features, combined with its intrinsic ability to utilise xylose, also makes this species a potentially interesting candidate for further research.

## Conclusions

The autoclave treated OMSW fibre evaluated in this study contained a large fraction of lignocellulosic sugars liberated through enzymatic hydrolysis to ~ 75% efficiency without additional pre-treatment. OMSW fibre hydrolysate was high in sugars but limited in microbially accessible nitrogen and phosphate. Marker inhibitor concentrations were relatively low and the majority of contaminating metals remained insoluble. We characterised growth of eight distinct species on nutrient-supplemented OMSW fibre hydrolysate and identified three top performers, *R. opacus* MITXM-61, *S. cerevisiae* ATCC200062 and *Z. mobilis* DSM424, which produced product at titres above ≥ 69% of theoretical yield. These diverse species are intrinsically well suited for growth on OMSW fibre hydrolysate and are promising candidates for industrial bioprocesses development. Overall, it was demonstrated that OMSW fibre has potential as a feedstock for producing renewable fuels and chemicals. Evaluating fermentation performance of candidate species in higher volume bioreactors and bioprocess LCAs are crucial future steps toward identifying which microbial platform would be most viable in an industrial MSW biorefinery.

## Materials and methods

### Production of organic fibre from municipal solid waste

The organic fraction of MSW was provided by Wilson Bio-Chemical in the form of Wilson Fibre^®^. A constructed batch of MSW was produced by combining a mixture of materials that reflected the composition of MSW produced in an average British household based on statistics published by the Department for Environment, Food and Rural Affairs (Additional file 1: Table S1) [23]. The constructed MSW mixture was subjected to pre-treatment in a pilot-scale Wilson System^®^. This involved autoclaving with dry steam at 160 °C and 72 psig for 45 min in a baffled vessel (50 kg capacity) rotating at 4 rpm. The pre-treated material was segregated into organic and inorganic fractions using manual sorting and sieving. The fibre was produced, homogenized and stored in ~ 1 kg bags at − 20 °C.

### Compositional Analysis of OMSW Fibre

For details of all compositional analyses see Additional file 1: Additional Methods.

### Enzymatic hydrolysis

OMSW fibre was defrosted and squeezed through synthetic cloth to reduce water content. The fibre was acidified to pH 5.0 with concentrated H_2_SO_4_ by manually massaging the acid into the fibre. Hydrolysis reactions were set up with a total dry solid loading of 20% w/v in 2 Litre conical flasks, mixed with water (pH 5.0, adjusted with concentrated H_2_SO_4_) and the lignocellulosic enzyme cocktail Cellic Ctec3 (Novozymes) (10% w/w enzymes to total available sugars). Hydrolysis was carried out for 48 h at 52.5 °C with shaking at 250 rpm. The resulting slurry was centrifuged (4000×*g*, 15 min) to separate the hydrolysate from un-hydrolysed solids. Hydrolysates from each flask were pooled and homogenized. Specific gravity was measured using a Brannan Specific Gravity Hydrometer (S50, 190 mm, Range: 1.000–1.050 SG). Hydrolysate was neutralised to pH 6.5 with concentrated KOH and frozen at − 20 °C. Sugar concentrations in the hydrolysate were measured by HPAEC as described in supplementary materials. Hydrolysis yields were calculated using the extended equation reported by [101].

### Hydrolysate sterilization

Hydrolysate was centrifugation (27,000×*g*, 30 min), the supernatant was vacuum filtered through a Buchner funnel with glass filter paper (Watman, GF/C) and then through a SteriCap Bottletop Filter Unit (0.22 µm PES membrane, 40 cm^2^ filtration area, 5–10 L capacity) (Merck-Millipore) in an aseptic laminar flow hood. The final sterile hydrolysate was aliquoted into 50 mL sterile conical tubes (Falcon) and stored at − 20 °C.

### Microorganisms, chemicals and media

All microorganisms used in this study are listed in Table 4 with their respective culture conditions, maintenance media and fermentation product of interest.

**Table 4:** Microorganisms, media and culture conditions used in this study

Species	Strain	T (°C)	Conditions	Medium^a^	Product of interest
*Clostridium saccharoperbutylacetonicum*	DSM14923	30	Anaerobic	RCM	Butanol
*Escherichia coli*	LW06	37	Aerobic/anaerobic	LB	Ethanol
*Geobacillus thermoglucosidasius*	DSM2542	55	Aerobic/anaerobic	TSB	Ethanol
*Pseudomonas putida*	NCIMB8249	30	Aerobic	LB	n/a
*Rhodococcus opacus*	MITXM-61	30	Aerobic	LB	Triacylglycerol
*Saccharomyces cerevisiae*	ATCC200062	30	Aerobic/anaerobic	YPD	Ethanol
*Schizosaccharomyces pombe*	JB953	32	Aerobic/anaerobic	YES	Ethanol
*Zymomonas mobilis*	DSM424	30	Aerobic/anaerobic	RM	Ethanol

^a^For details of each medium see Additional file 1

### Growth assays with *E. coli* LW06

OMSW fibre hydrolysate was supplementation with either K_2_HPO_4_, K_2_SO_4_, and NH_4_Cl_2_ (independently or in combination); with 1% vitamin-enriched yeast extract (VYE) (Sigma-Aldrich) or with MOPS defined medium. MOPS defined medium was based on Neidhardt’s MOPS defined medium [42] with some minor changes. MOPS defined medium contained (in mM): K_2_HPO_4_ (0.50), NH_4_Cl [10], MgCl_2_ (0.523), K_2_SO_4_ (0.276), FeSO_4_ (0.010), CaCl_2_ (5 × 10^−4^), NaCl [50], MOPS [40], (NH_4_)_6_(MO_7_)_24_ (3 × 10^−6^), H_3_BO_3_ (4 × 10^−4^) CoCl_2_ (3 × 10^−6^), CuSO_4_ (1 × 10^−5^), MnCl_2_ (8 × 10^−5^) and ZnSO_4_ (1 × 10^−5^). The last six components were prepared as a 5000× stock solution in 100 mL dH_2_O and stored at RT. Other stock solutions were prepared at the following concentrations in dH_2_O: 25% VYE; 10× MOPS defined medium; 2 M MOPS buffer (pH 7.0). Nutrient stocks were made up alone or in combination in 400 mM (10×) MOPS buffer with final concentrations of 5 mM K_2_HPO_4_, 3 mM K_2_SO_4_ and/or 100 mM NH_4_Cl_2_. All solutions were sterile filtered through a 0.22 µm syringe filter (Millex). In all experiments 9 mL MSW fibre hydrolysate was prepared in sterile 100 mL conical flasks with foam bungs and supplemented with nutrients, MOPS defined medium or VYE, depending on the experiment (see Fig. 2 for details). 100 µg/mL Ampicillin was always added. Overnight cultures were harvested in mid to late exponential phase and washed twice in dH_2_O before inoculating each flask to a starting OD_600_ of 0.01. OD_600_ was measured at regular intervals over 48 h.

### Fermentation medium

9.4 mL of sterile filtered OMSW fibre hydrolysate was supplemented with VYE (1% v/v, unless otherwise specified) and 40 mM MOPS buffer to a final volume of 10 mL. For aerobic and microaerobic fermentations the medium was transferred to sterile conical flasks (100 mL) and pre-heated to each species’ optimal temperature before inoculation. For anaerobic fermentations the medium was prepared in sterile wide-mouth conical flasks (250 mL) with foam bungs and allowed to deoxygenate in an anaerobic chamber for 4 days. Cysteine-HCl was then added to scavenge any residual oxygen. 10 mL was aliquoted to sterile anaerobic serum bottles (100 mL) by syringe.

### Fermentations

Fermentations were set up in triplicate with two negative controls each. Conical flasks (100 mL) used to grow *S. cerevisiae*, *G. thermoglucosidasius* and *E. coli* were sealed with airlocks to promote microaerobic conditions. Sterile airlocks were filled with sterile water before insertion under laminar flow. *R. opacus* and *P. putida* were grown in conical flasks (100 mL) with foam bungs to promote aeration. *Z. mobilis* and *C. saccharoperbutylacetonicum* were grown in anaerobic serum bottles (100 mL). Bottles were deoxygenated in anaerobic chambers for 1 week, sealed with silicone stoppers and crimp-tops and autoclaved. Overnight cultures of each species were harvested in mid exponential phase and washed twice in dH_2_O before re-suspending in fermentation medium to give a starting OD_600_ of 0.05. Cultures were incubated at each species’ optimal temperature with shaking at 160 rpm. The cultures were sampled at regular intervals over 48 h or 72 h for *R. opacus* and *P. putida*. Samples were used to measure OD_600_, final cell dry weight, sugar levels and levels of ethanol, butanol, acetone or triacylglycerol. Note that ethanol titres could have been higher than those measured in the fermentation liquid as some volatilisation into the flask headspace is expected under the experimental conditions. This is a notable limitation of our analysis methodology and shake flask screening in general. For detailed analytical methods and yield calculations see Additional file 1: Additional methods.

## Supplementary information

**Additional file 1: Table S1.** Composition of waste materials used for production of OMSW fibre on the Wilson Bio-Chemical Pilot Rig. A table showing the percentage composition of British MSW, based on estimates reported by the Department for Environment, Fisheries and Rural Affairs (DEFRA) [23] alongside the volumes of materials used to prepare a 20 kg batch of MSW that was used to produce the OMSW fibre for this project. **Table S2.** Percentage composition of constructed OMSW fibre. Provides a numerical break-down of the percentage composition data and standard deviations for all compositional analyses carried out on the OMSW fibre. **Table S3.** Concentrations of metals measured in constructed OMSW fibre. A list of all metals analysed in the OMSW fibre and their respective concentrations in mol/kg. **Figure S1.** Monosaccharide composition of hydrolysate produced from OMSW fibre. Provides the percentage and absolute concentration in g/L of monosaccharides measured in the OMSW fibre hydrolysate. **Figure S2.** Fractionation of metals after OMSW fibre hydrolysis. Shows the concentration of metals that were measured in the OMSW fibre hydrolysate liquid fraction and residual solids fraction. The theoretical levels of each metal that would be expected in a 20% total solids hydrolysis are also shown. **Additional Methods.**

## Data Availability

The datasets generated and/or analysed during the current study are available from the corresponding author on reasonable request. The datasets pertaining to the average composition of municipal solid waste in the United Kingdom that were used in this study are available from the Department for Environment, Fisheries and Rural Affairs (DEFRA), UK (Permalink: https://perma.cc/J3MD-SRQD).

## References

[1] Kaza S, Yao L, Bhada-Tata P, Van Woerden F (2018). What a waste 2.0: a global snapshot of solid waste management to 2050.

[2] Myhre G, Shindell D, Bréon F-M, Collins W, Fuglestvedt J, Huang J, Koch D, Lamarque J-F, Lee D, Mendoza B, Nakajima T, Robock A, Stephens G, Takemura T, Zhang H, Stocker TF, Qin D, Plattner G-K, Tignor M, Allen SK, Boschung J, Nauels A, Xia Y, Bex V, Midgley PM (2013). Anthropogenic and natural radiative forcing. Climate change 2013: the physical science basis, contribution of working group I to the fifth assessment report of the intergovernmental panel on climate change.

[3] EPA. Advancing Sustainable Materials Management: 2015 Fact Sheet. In: Agency USEP, editor. United States. 2018.

[4] EEA. municipal waste management across European countries. 2016.

[5] EEA. Managing municipal solid waste—a review of achievements in 32 European countries. In: (EEA) EEA, editor. 2/2013 ed. 2013.

[6] Lamb DT, Venkatraman K, Bolan N, Ashwath N, Choppala G, Naidu R (2014). Phytocapping: an alternative technology for the sustainable management of landfill sites. Crit Rev Environ Sci Technol..

[7] Department for Environment Food & Rural Afairs (DEFRA) (2018). Digest of waste and resource statistics.

[8] Marriott PE, Gomez LD, McQueen-Mason SJ (2016). Unlocking the potential of lignocellulosic biomass through plant science. New Phytol.

[9] Matsakas L, Gao Q, Jansson S, Rova U, Christakopoulos P (2017). Green conversion of municipal solid wastes into fuels and chemicals. Electron J Biotechnol.

[10] Barampouti EM, Mai S, Malamis D, Moustakas K, Loizidou M (2019). Liquid biofuels from the organic fraction of municipal solid waste: a review. Renew Sustain Energy Rev.

[11] Abdullah J, Greetham D (2016). Optimizing cellulase production from municipal solid waste (MSW) using solid state fermentation (SSF). J Fund Renew Energy Appl..

[12] Lay J-J, Lee Y-J, Noike T (1999). Feasibility of biological hydrogen production from organic fraction of municipal solid waste. Water Res.

[13] Aiello-Mazzarri C, Agbogbo FK, Holtzapple MT (2006). Conversion of municipal solid waste to carboxylic acids using a mixed culture of mesophilic microorganisms. Bioresour Technol.

[14] Adhikari BK, Trémier A, Barrington S, Martinez JJW (2013). Valorization B. Biodegradability of municipal organic waste: a respirometric test. Waste Biomass Valoriz.

[15] Farmanbordar S, Karimi K, Amiri H (2018). Municipal solid waste as a suitable substrate for butanol production as an advanced biofuel. Energy Convers Manag..

[16] Ghanavati H, Nahvi I, Karimi K (2015). Organic fraction of municipal solid waste as a suitable feedstock for the production of lipid by oleaginous yeast *Cryptococcus aerius*. Waste Manag.

[17] Jensen JW, Felby C, Jørgensen H (2011). Cellulase hydrolysis of unsorted MSW. Appl Biochem Biotechnol.

[18] Hartmann H, Ahring BK (2005). Anaerobic digestion of the organic fraction of municipal solid waste: influence of co-digestion with manure. Water Res.

[19] Lavagnolo MC, Girotto F, Rafieenia R, Danieli L, Alibardi L (2018). Two-stage anaerobic digestion of the organic fraction of municipal solid waste—effects of process conditions during batch tests. Renew Energy.

[20] McCaskey TA, Zhou SD, Britt SN, Strickland R (1994). Bioconversion of municipal solid waste to lactic acid by Lactobacillus species. Appl Biochem Biotechnol.

[21] Ma HZ, Wang QH, Qian DY, Gong LJ, Zhang WY (2009). The utilization of acid-tolerant bacteria on ethanol Production from kitchen garbage. Renew Energy.

[22] Dang Y, Sun D, Woodard TL, Wang L-Y, Nevin KP, Holmes DE (2017). Stimulation of the anaerobic digestion of the dry organic fraction of municipal solid waste (OFMSW) with carbon-based conductive materials. Bioresour Technol..

[23] DEFRA. Digest of Waste and Resource Statistics—2015 Edition. Department for Environment, Food and Rural Affairs, Department for Food EaRA; 2015 January 2015. Contract No.: PB14292.

[24] WilsonBio-Chemical. The Autoclave: Process Overview 2018. http://wilsonbio-chemical.co.uk/the-wilson-system/.

[25] Meng F, Ibbett R, de Vrije T, Metcalf P, Tucker G, McKechnie J (2019). Process simulation and life cycle assessment of converting autoclaved municipal solid waste into butanol and ethanol as transport fuels. Waste Manag.

[26] Rumbold K, van Buijsen HJJ, Gray VM, van Groenestijn JW, Overkamp KM, Slomp RS (2010). Microbial renewable feedstock utilization: a substrate-oriented approach. Bioeng Bugs..

[27] Rumbold K, van Buijsen HJ, Overkamp KM, van Groenestijn JW, Punt PJ, van der Werf MJ (2009). Microbial production host selection for converting second-generation feedstocks into bioproducts. Micorb Cell Fact.

[28] Lau MW, Gunawan C, Balan V, Dale BE (2010). Comparing the fermentation performance of *Escherichia coli* KO11, *Saccharomyces cerevisiae* 424A(LNH-ST) and *Zymomonas mobilis* AX101 for cellulosic ethanol production. Biotechnol Biofuels..

[29] Puri DJ, Heaven S, Banks CJ (2013). Improving the performance of enzymes in hydrolysis of high solids paper pulp derived from MSW. Biotechnol Biofuels..

[30] Ballesteros M, Sáez F, Ballesteros I, Manzanares P, Negro MJ, Martínez JM (2010). Ethanol production from the organic fraction obtained after thermal pretreatment of municipal solid waste. Appl Biochem Biotechnol.

[31] Mahmoodi P, Karimi K, Taherzadeh MJ (2018). Hydrothermal processing as pretreatment for efficient production of ethanol and biogas from municipal solid waste. Bioresour Technol.

[32] Farmanbordar S, Amiri H, Karimi K (2018). Simultaneous organosolv pretreatment and detoxification of municipal solid waste for efficient biobutanol production. Bioresour Technol.

[33] Sluiter A, Ruiz R, Scarlata C, Sluiter J, Templeton D. Determination of extractives in biomass—laboratory analytical procedure. National Renewable Energy Laboratories (NREL); 2008. Contract No.: Technical Report NREL/TP-510-42619.

[34] Zaldivar J, Ingram LO (1999). Effect of organic acids on the growth and fermentation of ethanologenic *Escherichia coli* LY01. Biotechnol Bioeng..

[35] Chun AY, Yunxiao L, Ashok S, Seol E, Park S (2014). Elucidation of toxicity of organic acids inhibiting growth of *Escherichia coli* W. Biotechnol Bioprocess Eng.

[36] Hou J, Ding C, Qiu Z, Zhang Q, Xiang W-N (2017). Inhibition efficiency evaluation of lignocellulose-derived compounds for bioethanol production. J Clean Prod..

[37] Zaldivar J, Martinez A, Ingram LO (1999). Effect of selected aldehydes on the growth and fermentation of ethanologenic *Escherichia coli*. Biotechnol Bioeng..

[38] Kalantari N. Evaluation of toxicity of heavy metals for *Escherichia coli* growth. 2008.

[39] Nies DH (1999). Microbial heavy-metal resistance. Appl Microbiol Biotechnol.

[40] Majtan T, Frerman FE, Kraus JP (2011). Effect of cobalt on *Escherichia coli* metabolism and metalloporphyrin formation. Biometals.

[41] Bird LJ, Coleman ML, Newman DK (2013). Iron and copper act synergistically to delay anaerobic growth of bacteria. Appl Environ Microbiol.

[42] Neidhardt FC, Bloch PL, Smith DF (1974). Culture medium for enterobacteria. J Bacteriol.

[43] Jørgensen H (2009). Effect of nutrients on fermentation of pretreated wheat straw at very high dry matter content by *Saccharomyces cerevisiae*. Appl Biochem Biotechnol.

[44] Kampen WH, Vogel HC, Todaro CM (2014). Chapter 4—Nutritional requirements in fermentation processes. Fermentation and biochemical engineering handbook.

[45] Cripps RE, Eley K, Leak DJ, Rudd B, Taylor M, Todd M (2009). Metabolic engineering of *Geobacillus thermoglucosidasius* for high yield ethanol production. Metab Eng.

[46] Sheng L, Kovács K, Winzer K, Zhang Y, Minton NP (2017). Development and implementation of rapid metabolic engineering tools for chemical and fuel production in Geobacillus thermoglucosidasius NCIMB 11955. Biotechnol Biofuels.

[47] Belda E, van Heck RGA, Lopez-Sanchez MJ, Cruveiller S, Barbe V, Fraser C (2016). The revisited genome of *Pseudomonas putida* KT2440 enlightens its value as a robust metabolic chassis. Environ Microbiol.

[48] Woodruff LB, May BL, Warner JR, Gill RT (2013). Towards a metabolic engineering strain “commons”: an *Escherichia coli* platform strain for ethanol production. Biotechnol Bioeng.

[49] Hayles J, Nurse P (1992). Genetics of the fission yeast *Schizosaccharomyces Pombe*. Annu Rev Genet.

[50] Choi GW, Um HJ, Kim M, Kim Y, Kang HW, Chung BW (2010). Isolation and characterization of ethanol-producing *Schizosaccharomyces pombe* CHFY0201. J Microbiol Biotechnol.

[51] Tura A, Fontana RC, Camassola MJAB (2018). *Schizosaccharomyces pombe* as an efficient yeast to convert sugarcane bagasse pretreated with ionic liquids in ethanol. Appl Biochecm Biotechnol.

[52] Benito Á, Jeffares D, Palomero F, Calderón F, Bai F-Y, Bähler J (2016). Selected *Schizosaccharomyces pombe* strains have characteristics that are beneficial for winemaking. PLoS ONE.

[53] Jeffries TW, Kurtzman CP (1994). Strain selection, taxonomy, and genetics of xylose-fermenting yeasts. Enzyme Microb Technol..

[54] Bucholz SE, Dooley MM, Eveleigh DE (1987). Zymomonas—an alcoholic enigma. Tibtech..

[55] Young E, Lee SM, Alper H (2010). Optimizing pentose utilization in yeast: the need for novel tools and approaches. Biotechnol Biofuels.

[56] Novy V, Wang R, Westman JO, Franzén CJ, Nidetzky B (2017). *Saccharomyces cerevisiae* strain comparison in glucose–xylose fermentations on defined substrates and in high-gravity SSCF: convergence in strain performance despite differences in genetic and evolutionary engineering history. Biotechnol Biofuels.

[57] Agrawal M, Mao Z, Chen RR (2010). Adaptation yields a highly efficient xylose-fermenting *Zymomonas mobilis* strain. Biotechnol Bioeng.

[58] Spindler DD, Wyman CE, Grohmann K, Mohagheghi A (1989). Simultaneous saccharification and fermentation of pretreated wheat straw to ethanol with selected yeast strains and beta-glucosidase supplementation. Appl Biochem Biotechnol.

[59] Nguyen TY, Cai CM, Kumar R, Wyman CE (2017). Overcoming factors limiting high-solids fermentation of lignocellulosic biomass to ethanol. Proc Natl Acad Sci USA.

[60] Smith J, van Rensburg E, Gorgens JF (2014). Simultaneously improving xylose fermentation and tolerance to lignocellulosic inhibitors through evolutionary engineering of recombinant *Saccharomyces cerevisiae* harbouring xylose isomerase. BMC Biotechnol..

[61] Guner FS, Yagci Y, Erciyes AT (2006). Polymers from triglyceride oils. Prog Polym Sci.

[62] Desai JD, Banat IM (1997). Microbial production of surfactants and their commercial potential. Microbiol Mol Biol Rev.

[63] Sawpan MA (2018). Polyurethanes from vegetable oils and applications: a review. J Polym Res.

[64] Kurosawa K, Wewetzer SJ, Sinskey AJ (2014). Triacylglycerol production from corn stover using a xylose-fermenting *Rhodococcus opacus* strain for lignocellulosic biofuels. J Microb Biochem Technol..

[65] Alvarez HM, Silva RA, Herrero M, Hernández MA, Villalba MS (2013). Metabolism of triacylglycerols in Rhodococcus species: insights from physiology and molecular genetics. J Mol Biochem..

[66] Alvarez HM, Mayer F, Fabritius D, Steinbuchel A (1996). Formation of intracytoplasmic lipid inclusions by *Rhodococcus opacus* strain PD630. Arch Microbiol.

[67] Klopfenstein WE (1982). Estimation of cetane index for esters of fatty-acids. J Am Oil Chem Soc.

[68] Giakoumis EG (2013). A statistical investigation of biodiesel physical and chemical properties, and their correlation with the degree of unsaturation. Renew Energy.

[69] CEN. EN 590:2009, Automotive fuels—diesel-requirements and test methods. In: (CEN) ECfS, editor. 2009.

[70] Jiménez-Díaz L, Caballero A, Pérez-Hernández N, Segura A (2016). Microbial alkane production for jet fuel industry: motivation, state of the art and perspectives. Microb Biotechnol.

[71] Quiros R, Gabarrell X, Villalba G, Barrena R, Garcia A, Torrente J (2015). The application of LCA to alternative methods for treating the organic fiber produced from autoclaving unsorted municipal solid waste: case study of Catalonia. J Clean Prod..

[72] Wojnowska-Baryła I, Kulikowska D, Bernat K, Kasiński S, Zaborowska M, Kielak T (2019). Stabilisation of municipal solid waste after autoclaving in a passively aerated bioreactor. Waste Manag Res.

[73] Jönsson LJ, Martin C (2016). Pretreatment of lignocellulose: formation of inhibitory by-products and strategies for minimizing their effects. Bioresour Technol.

[74] Jensen JW, Felby C, Jorgensen H, Ronsch GO, Norholm ND (2010). Enzymatic processing of municipal solid waste. Waste Manag.

[75] Mahmoodi P, Karimi K, Taherzadeh MJ (2018). Efficient conversion of municipal solid waste to biofuel by simultaneous dilute-acid hydrolysis of starch and pretreatment of lignocelluloses. Energy Convers Manag..

[76] Li S, Zhang X, Andresen JM (2012). Production of fermentable sugars from enzymatic hydrolysis of pretreated municipal solid waste after autoclave process. Fuel.

[77] Modenbach AA, Nokes SE (2012). The use of high-solids loadings in biomass pretreatment-a review. Biotechnol Bioeng.

[78] Almeida JRM, Bertilsson M, Gorwa-Grauslund MF, Gorsich S, Liden G (2009). Metabolic effects of furaldehydes and impacts on biotechnological processes. Appl Microbiol Biotechnol.

[79] Kerby C, Vriesekoop F (2017). An overview of the utilisation of brewery by-products as generated by British craft breweries. Beverages..

[80] Liu HH, Zhang J, Yuan J, Jiang XL, Jiang LY, Zhao G (2019). Omics-based analyses revealed metabolic responses of *Clostridium acetobutylicum* to lignocellulose-derived inhibitors furfural, formic acid and phenol stress for butanol fermentation. Biotechnol Biofuels..

[81] Field JA, Lettinga G (1992). Toxicity of tannic compounds to microorganisms. Plant Polyphen..

[82] Gyulev IS, Willson BJ, Hennessy RC, Krabben P, Jenkinson ER, Thomas GH (2018). Part by part: synthetic biology parts used in *Solventogenic clostridia*. ACS Synth Biol..

[83] Henson WR, Hsu F-F, Dantas G, Moon TS, Foston M (2018). Lipid metabolism of phenol-tolerant *Rhodococcus opacus* strains for lignin bioconversion. Biotechnol Biofuels.

[84] Petrovič U (2015). Next-generation biofuels: a new challenge for yeast. Yeast.

[85] Panesar PS, Marwaha SS, Kennedy JF (2006). *Zymomonas mobilis*: an alternative ethanol producer. J Chem Technol Biotechnol.

[86] Dien BS, Cotta MA, Jeffries TW (2003). Bacteria engineered for fuel ethanol production: current status. Appl Microbiol Biotechnol..

[87] Waeltermann M, Luftmann H, Baumeister D, Kalscheuer R, Steinbuchel A (2000). Rhodococcus opacus strain PD630 as a new source of high value single cell oil? Isolation and characterization of triacylglycerol and other storage lipids. Microbiology.

[88] Kurosawa K, Boccazzi P, de Almeida NM, Sinskey AJ (2010). High-cell-density batch fermentation of *Rhodococcus opacus* PD630 using a high glucose concentration for triacylglycerol production. J Biotechnol.

[89] Kurosawa K, Wewetzer SJ, Sinskey AJ (2013). Engineering xylose metabolism in triacylglycerol-producing *Rhodococcus opacus* for lignocellulosic fuel production. Biotechnol Biofuels.

[90] Fei Q, Wewetzer SJ, Kurosawa K, Rha C, Sinskey AJ (2015). High-cell-density cultivation of an engineered *Rhodococcus opacus* strain for lipid production via co-fermentation of glucose and xylose. Process Biochem.

[91] Büchs J (2001). Introduction to advantages and problems of shaken cultures. Biochem Eng J.

[92] Neubauer P, Cruz N, Glauche F, Junne S, Knepper A, Raven M (2013). Consistent development of bioprocesses from microliter cultures to the industrial scale. Eng Life Sci.

[93] Julio R, Albet J, Vialle C, Vaca-Garcia C, Sablayrolles C (2017). Sustainable design of biorefinery processes: existing practices and new methodology. Biofuels Bioprod Biorefining.

[94] Wältermann M, Hinz A, Robenek H, Troyer D, Reichelt R, Malkus U (2004). Mechanism of lipid-body formation in prokaryotes: how bacteria fatten up. Mol Microbiol.

[95] Wang C, Chen L, Rakesh B, Qin Y, Lv R (2012). Technologies for extracting lipids from oleaginous microorganisms for biodiesel production. Front Energy..

[96] Jung YK, Kim TY, Park SJ, Lee SY (2010). Metabolic engineering of *Escherichia coli* for the production of polylactic acid and its copolymers. Biotechnol Bioeng.

[97] Park SY, Yang D, Ha SH, Lee SY (2018). Metabolic engineering of microorganisms for the production of natural compounds. Adv Biosyst.

[98] Wang C, Pfleger BF, Kim S-W (2017). Reassessing *Escherichia coli* as a cell factory for biofuel production. Curr Opin Biotechnol..

[99] Nikel PI, de Lorenzo V (2014). Robustness of *Pseudomonas putida* KT2440 as a host for ethanol biosynthesis. New Biotechnol..

[100] Wang HH, Zhou XR, Liu QA, Chen GQ (2011). Biosynthesis of polyhydroxyalkanoate homopolymers by *Pseudomonas putida*. Appl Microbiol Biotechnol.

[101] Kristensen JB, Felby C, Jørgensen H (2009). Yield-determining factors in high-solids enzymatic hydrolysis of lignocellulose. Biotechnol Biofuels.

