# Supplementary material for: Robust microorganisms for biofuel and chemical production from municipal solid waste
Source: Microb Cell Fact. 2020 Mar 16;19:68. doi: 10.1186/s12934-020-01325-0 (PMC7077162; doi:10.1186/s12934-020-01325-0)
Supplement: Supplementary file 1 — Additional file 1: Table S1. Composition of waste materials used for production of OMSW fibre on the Wilson Bio-Chemical Pilot Rig. A table showing the percentage composition of British MSW, based on estimates reported by the Department for Environment, Fisheries and Rural Affairs (DEFRA) [23] alongside the volumes of materials used to prepare a 20 kg batch of MSW that was used to produce the OMSW fibre for this project. Table S2. Percentage composition of constructed OMSW fibre. Provides a numerical break-down of the percentage composition data and standard deviations for all compositional analyses carried out on the OMSW fibre. Table S3. Concentrations of metals measured in constructed OMSW fibre. A list of all metals analysed in the OMSW fibre and their respective concentrations in mol/kg. Figure S1. Monosaccharide composition of hydrolysate produced from OMSW fibre. Provides the percentage and absolute concentration in g/L of monosaccharides measured in the OMSW fibre hydrolysate. Figure S2. Fractionation of metals after OMSW fibre hydrolysis. Shows the concentration of metals that were measured in the OMSW fibre hydrolysate liquid fraction and residual solids fraction. The theoretical levels of each metal that would be expected in a 20% total solids hydrolysis are also shown. Additional Methods. [file 12934_2020_1325_MOESM1_ESM.pdf]

Additional File 1: Tables, Figures and Methods

Aritha Dornau[a], James F. Robson[b], Gavin H. Thomas[b,c] & Simon J. McQueen-Mason[a,c]*

[a]Centre for Novel Agricultural Products (CNAP), Department of Biology, University of York, Heslington, York, YO10 5DD, United Kingdom.

[b]Department of Biology, University of York, Heslington, York, YO10 5DD, United Kingdom.

[c] Shared senior authorship.

*Corresponding author: Simon McQueen-Mason, Tel: +44 1904 328775, e-mail: simon.mcqueenmason@york.ac.uk

Table S1: Composition of waste materials used for production of OMSW fibre on the Wilson Bio-Chemical Pilot Rig.

**Masses are based on the percentage composition of the major MSW components reported by DEFRA (23) and adjusted to 100%.*

W.E.E.E. = Waste Electrical and Electronic Equipment.

Note: some inorganic components were not included in the constructed MSW for health and safety reasons or because these larger items would typically be sorted out prior to autoclaving.

Type of waste	DEFRA reported composition (%)	Adjusted to 100%	Per 20 Kg batch (Kg)*
Food	15.0	20.80	4.16
Plastic film	8.9	12.34	2.47
Dense plastic	11.3	15.67	3.13
Paper	10.5	14.56	2.91
Card	8.4	11.65	2.33
Wood	7.6	10.54	2.11
Metal	3.5	4.85	0.97
Garden	2.7	3.74	0.75
Other organic	1.7	2.36	0.47
W.E.E.E.	1.5	2.08	0.42
Hazardous/batteries	1.0	1.39	0.28
Carpet/underlay/furniture	6.0	-	-
Brick/plaster/soil	5.9	-	-
Textiles/shoes	5.7	-	-
Fines	2.5	-	-
Glass	2.2	-	-
Sanitary	2.0	-	-
Other non-combustible	1.6	-	-
**Total**	**100**	**100.00**	**20.00**
**Estimated Biodegradability**	**51.4**	**63.65**	**12.73**

Table S2: Percentage composition of constructed OMSW fibre

Components were measured by a range of established methods. For details see materials and methods in paper. Oil and protein were measured separately but are ethanol and water soluble, respectively, and are thus shown as a fraction of non-structural components extracted by water or ethanol. A breakdown of the monosaccharides measured in the hemicellulose fraction are given as percentages of the total dry weight of fibre. All data are averages of at least triplicate analyses.
Glu = Glucose; Xyl = Xylose; Man = Mannose; Fuc = Fucose; Ara = Arabinose; Rha = Rhamnose; Gal = Galactose; GalA = Galacturonic acid. n/a = not applicable.

Component	Percentage of total dry mass (%)	±SD
**Cellulose**	37.61	2.73
**Hemicellulose**		
Fuc	*0.02*	*0.002*
Ara	*0.27*	*0.02*
Rha	*0.06*	*0.002*
Gal	*0.41*	*0.02*
Glu	*1.10*	*0.08*
Xyl	*1.34*	*0.03*
Man	*0.95*	*0.10*
GalA	*0.10*	*0.02*
**Total:**	**4.25**	**0.14**
**Lignin**	15.78	0.37
**Ash**	14.74	1.47
**Metals**	1.33	0.26
**Extractives (water)**		
Protein	*3.23*	*0.40*
Extractives (other)	*5.92*	*n/a*
**Total:**	**9.15**	**2.62**
**Extractives (ethanol)**		
Oil	*1.72*	*0.25*
Extractives (other)	*6.23*	*n/a*
**Total:**	**7.95**	**0.92**
**SUM:**	90.83	4.21
**Other (remaining):**	9.17	*n/a*

Table S3: Concentration of metals measured in constructed OMSW fibre

Metals were measured by ionisation coupled plasma mass spectrometry (ICPMS) against Agilent certified multi-element environmental calibration standard No. 5183-4688. Note that units change as concentrations range from mol/Kg to µmol/Kg.
±SD *= Standard deviation of triplicates.*
n/d *= Not detected.*

Metal	Concentration	±SD	Units
**Ca^{43}**	1.03	0.12	mol/Kg
**Ca^{44}**	1.06	0.14	mol/Kg
**Al**	166.90	27.50	mmol/Kg
**K**	66.53	6.44	mmol/Kg
**Fe**	62.44	28.48	mmol/Kg
**Na**	57.48	5.43	mmol/Kg
**Mg**	44.44	6.00	mmol/Kg
**Zn**	2.47	1.65	mmol/Kg
**Mn**	0.76	0.06	mmol/Kg
**Ni**	0.33	0.25	mmol/Kg
**Cu**	0.33	0.06	mmol/Kg
**Ba**	0.16	0.02	mmol/Kg
**Cr**	0.11	0.01	mmol/Kg
**V**	0.04	0.01	mmol/Kg
**Pb**	0.01	0.00	mmol/Kg
**Sb**	0.01	0.00	mmol/Kg
**V**	39.98	5.08	µmol/Kg
**Pb**	13.77	3.10	µmol/Kg
**Sb**	10.39	0.63	µmol/Kg
**Co**	9.29	0.87	µmol/Kg
**As**	1.20	0.66	µmol/Kg
**U**	0.46	0.09	µmol/Kg
**Cd**	0.30	0.09	µmol/Kg
**Tl**	0.03	0.02	µmol/Kg
**Th**	*n/d*	*n/d*	-
**Mo**	*n/d*	*n/d*	-
**Ag**	*n/d*	*n/d*	-

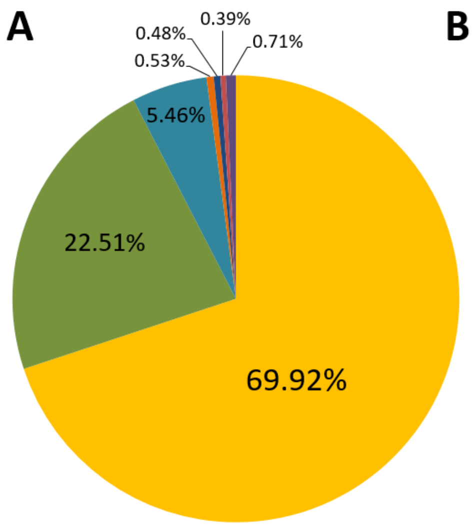

B

Sugars (g/L)		±SD
Glu	34.39	±1.31
Xyl	17.51	±1.27
Man	4.25	±0.613
Fuc	0.41	±0.32
Ara	0.37	±0.27
Rha	0.31	±0.30
Gal	0.56	±0.38
**Total**	**58.24**	**±5.76**

Figure S1: Monosaccharide composition of hydrolysate produced from OMSW fibre

A: *Percentage abundance of sugars in hydrolysate of MSW fibre after 48 hours hydrolysis with Cellic Ctec3 (Novozymes).* ***B:*** *Quantitative concentration of sugars in the hydrolysate (average of triplicate measurements with standard deviation from the mean (±SD)).*

Colours in ***A*** *correspond to the colours marked for each sugar in* ***B****.*

Glu = Glucose; Xyl = Xylose; Man = Mannose; Fuc = Fucose; Ara = Arabinose; Rha = Rhamnose; Gal = Galactose.

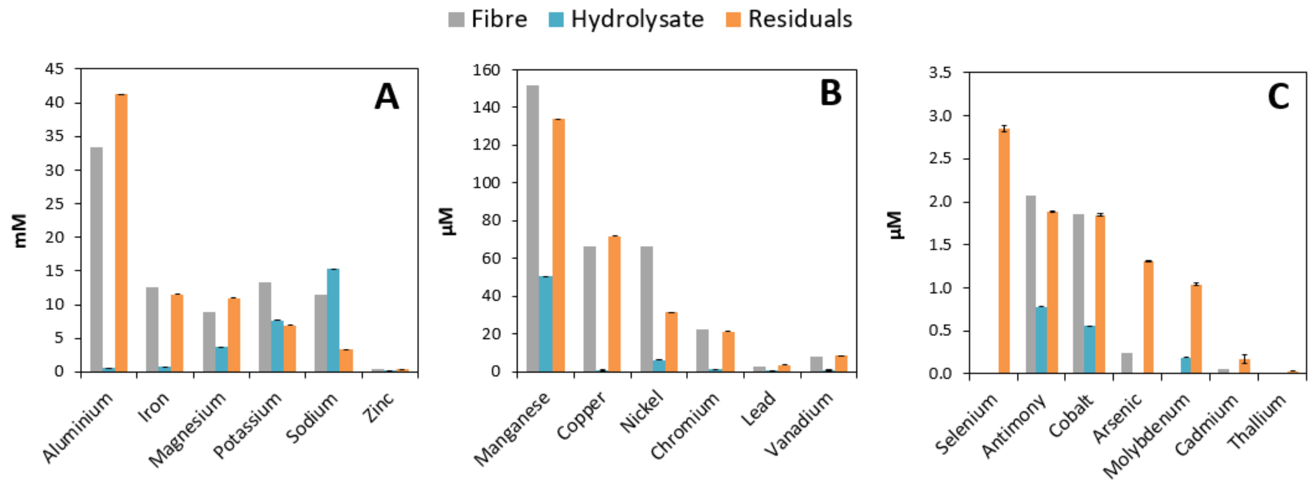

Figure S2: Fractionation of metals after OMSW fibre hydrolysis

*'**Fibre**' (grey bars): Calculated concentration of metals that would be released into the hydrolysate through enzymatic hydrolysis, assuming 100% solubilisation. These values are based on the metal levels measured in the OMSW fibre prior to hydrolysis and account for a five-fold dilution in a 20% total solids hydrolysis reaction (see sup. Table 2). '**Hydrolysate**' (Blue bars): The concentration of metals measured in the MSW fibre hydrolysate liquid fraction after hydrolysis. '**Residuals**' (orange bars): The concentration of metals measured in the residual solid material left over after hydrolysis. Calculations accounted for a 5-fold dilution in a 20% total solids hydrolysis reaction.*

Additional Methods

Moisture content, extractives and ash content were determined according to standard protocols developed by the National Renewable Energy Laboratory (NREL) (2-4). Other compositional methods, described below, were carried out in triplicate using OMSW fibre dried at 45°C and ball milled on a Retsch TissueLyser II (Quiagen).

Composition of lignocellulosic fraction. Lignocellulose composition was determined as described previously (5). Briefly, 4 mg of OMSW fibre was hydrolysed with trifluoroacetic acid to isolate all hemicellulosic sugars as monosaccharides and then quantified by high-performance anion exchange chromatography (HPAEC) as described below. The pellet from hemicellulose analysis was subjected to a modified Seaman hydrolysis to hydrolyse all crystalline cellulose, then quantified by a colourimentric anthrone assay. Lignin was analysed by the acetyl bromide method according to Fukushima and Hatfield (6).

Oil. Samples were subjected to continuous extraction with ether. All extracted oil was dried and weighed. Residual solid material was boiled with hydrochloric acid to release any bound fats, filtered through filter paper and washed repeatedly with water until neutralised. The filter paper was dried and any retained fat was again extracted with ether, dried and weighed. The total oil was reported as the sum of oil measured in the first and second ether extraction.

Protein. Samples were subjected to the Dumas method (7) with a Leco FP285 Nitrogen Analyser.

Metals. Samples were digested using 1:1 nitric:sulfuric acid (trace metal grade) in a PTFE digestion vessel. Vessels were transferred to a microwave digestion system (Ethos Up) and heated to 200°C for 15 minutes, cooled to room temperature, transferred to a volumetric flask (100 ml) and diluted to volume with dH_2O. Samples were further diluted 100-fold with dH_2O and analysed on an Agilent 7700x inductively coupled plasma-mass spectrometer (ICP-MS). Results were quantified against seven calibration standards (Agilent certified multi-element environmental calibration standard No. 5183-4688). Calibration curves had R^2 values of $\geq$0.998. dH_2O was used as a blank.

UPLC-MS. Furfural, 5-Hydroxymethylfurfural (5-HMF), vanillin and levulinic acid were measured in the OMSW fibre hydrolysate by ultra-performance liquid chromatography (UPLC) with mass spectrometric (MS) detection. Chromatographic separation was carried out on a Waters Acquity I-Class System with a VanGuard pre-column with C18 frit (Waters) and a BEH C18 column (100x2.1mm, 1.7 µm) (Waters). Each run was performed with a gradient (min/%B = 0/16, 2.5/16, 2.8/100, 2.9/100, 3.3/16, 4/16) of solvents A (5% MeOH, 0.1% acetic acid) and B (0.1% acetic acid in MeOH). Injection volume was 2 µl with a flow rate of 0.5 ml/min at 45°C. MS was carried out on a Thermo Endura Triple Quad with HESI positive ion source and single reaction monitoring (SRM) with one transition for each compound (compound, precursor *m/z*/product*m/z*: levulinic acid, 99.12/71.22; HMF, 109.09/81.15; furfural, 97.12/69.22; vanillin, 153.05/93.11). Data was analysed with Thermo Xcalibur 4.0.27.10 software.

GC-FID. A range of organic acids were measured in the hydrolysate by gas chromatography (GC) with flame-ionization detection (FID) on a Nukol column (30 m x 0.25 mm, I.D 0.25 µm (24107)) with a helium carrier (30 psi) and liquid injection. Detectors and injectors were operated at 200°C. Temperature was increased from 75-150°C (10°C/min), 150-200°C (20°C/min) and held for 10 minutes. Hydrolysate was prepared for analysis in triplicate by acidifying 1 ml with 7.5 µl concentrated orthophosphoric acid (Sigma-Aldrich) and run in parallel alongside a volatile free acid standard (CRM46975, Sigma-Aldrich).

Microorganisms, Chemicals and Media

Reinforced Clostridial medium (RCM), Luria-Bertani (LB) medium and Tryptic soy broth (TSB) were purchased from Thermo Scientific (Oxoid). Rich medium (RM) contained (per Litre) D-glucose (20 g), Yeast extract (10 g) and K2HPO4 (2 g). Yeast extract peptone dextrose (YPD) contained (per Litre) D-glucose (20 g), Yeast extract (5 g) and Bacto peptone (10 g). Yeast extract with supplements (YES) contained (per Litre) Yeast extract (5 g), D-glucose (30 g) and supplements (225 mg adenine, histidine, leucine, uracil and lysine hydrochloride). All components were purchased from Sigma-Aldrich. All

species were stored in 25% glycerol at -80°C and were maintained at 4°C on 15% agar plates of their respective media.

Final cell dry weight. At the end of fermentation 1 ml of each culture was transferred to dry, pre-weighed Microtubes (1.5 ml, Eppendorf). The samples were centrifuged (4000 x g, 5 minutes) and then washed twice in water. For *R. opacus* MITXM-61 the entire culture was transferred to dry, pre-weighed 15 ml conical tubes (Falcon^{TM}) and subjected to the same centrifugation and washing steps described above. All cell pellets were frozen at -20°C and then lyophilised in a Heto PowerDry LL3000 freeze dryer. The final mass of the dry cell material was used to calculate the final dry cell weight produced per ml of fermentation broth. The dry cell material from *R. opacus* fermentations was used for fatty acid profiling as described below.

HPAEC. Frozen hydrolysates and fermentation samples were thawed and centrifuged (4000 x g, 5 minutes). 10 µl of each supernatant was serially diluted 1:250 with dH_2O and analysed by high-performance anion exchange chromatography (HPAEC) on an ICS-3000 PAD system with an electrochemical gold electrode using a Dionex CarboPac PA20 analytical column (3x150 mm) and guard column (3x30 mm). Identification and quantification of glucose and xylose was carried out by comparing retention times and integrated peak areas of the samples to an equimolar standard mixture of monosaccharides during the same run under the same conditions.

Ethanol analysis. Ethanol concentration was measured over the course of fermentation as follows: 10 µl of fermentation supernatant was mixed with 500 µl of 1M NaCl with 0.0004% propan-1-ol internal standard in 2ml crimp-top flat-bottom GC vials. Samples were analysed on an Agilent 6890 Gas Chromatograph (GC) fitted with a Gerstel Multi-purpose 2 (MPS2) autosampler with SPME pink fibre (23-Gauge, 65µm, PDMS/DVB, SUPELCO) linked to a LECO Pegasus IV Time of Flight (TOF) Mass Spectrometer (MS). The GC was fitted with a glass injector liner (Ultra Inert, Straight 0.75mm ID 5pk, Restek), Rxi5Sil Column with Integra guard (Restek) and was operated with an initial temperature of 70°C (2.5 min. hold) then ramped at 65°C/min. to 200°C and held for 1 minute before cooling at

70°C/min. to 70°C (1 min. hold). The MS mass range was 10-300 with an electron energy of -70V. The autosampler was run for 10 minutes, including cooling time (1 minute extraction, 0.1 minute desorb and 7 minutes fibre bakeout). Results were quantified against the internal standard and a standard curve of 0.1-4.0% v/v ethanol and then converted to g/L using the density of ethanol (0.7893 g/cm^3).

Fatty acid profiling. To determine the fatty acid composition 10 ±0.05 mg of freeze-dried *R. wratislavensis* MITXM-61 cells were spiked with 10 µl of 25 mg/ml Heneicosanoic acid (21:0) internal standard (part no. H5149, Sigma) and then transmethylated to fatty acid methyl esters (FAMES) with 500 µl 1N Methanolic HCl and 200 µl Hexane at 85°C for 24 hours. Samples were cooled to 25°C, mixed with 250 µl 0.9% KCl and 600 ul Hexane and vortexed. 100µl of the upper hexane layer was aspirated to a tapered vial with crimp-cap lid and analysed with a Thermo Trace GC Ultra GC-FID (Gas Chromatograph with Flame Ionisation Detector) on a SGE BPX70 column (10M x 0.1 mm, part no. 054600).

Calculating the Cetane Number (CN). A cetane index (CN_i) was calculated for each individual FAME using **Equation 1** and then the CN for the total FAME mixture extracted from *R. opacus* was calculated using **Equation 2**.

Equation 1: Calculating the cetane index for each FAME

$$\boldsymbol{CN_i} = 58.1 + 2.8 \times \left(\frac{n_i - 8}{2}\right) - 15.9 \times db_i$$

Where:

CN_i = *The cetane number index of the FAME, dimensionless.*

n_i = *The number of carbon bonds in the FAME molecule (between 8 – 24)*

db_i = *The number of double bonds in the FAME molecule, dependent upon saturation*

58.1 = *The cetane index for the shortest FAME within the scope of the equation (C8:00)*

2.8 = *Cetane index increment when the FAME chain is increased by two carbon atoms*

15.9 = *Cetane index increment when a double bond is present within the FAME molecule*

Equation 2: Calculating the cetane number for a FAME mixture

$$\boldsymbol{CN} = \sum_i w_i CN_i$$

Where:

CN = *The cetane number for the total mixture of FAMEs, dimensionless.*

CN_i = *The cetane index of an individual FAME, calculated with* ***Equation 4.9***

W_i = *The mass percentage of an individual FAME within the FAME mixture, %w/w*

Calculating Fermentation Yield Parameters. To quantitatively evaluate the fermentation efficiency of each species key yield parameters were calculated for each fermentation, including the percentage of total fermentable sugars consumed over the course of fermentation (**Equation 3**), the product to substrate ratio (i.e. specific yield) (**Equation 4**), the total product yield expressed as a percentage of the theoretical maximum (**Equation 5**) and the overall productivity in g/L.h^{-1} (i.e. process productivity) (**Equation 6**). The yield per tonne (**Equation 7**) was calculated using either the glucose and xylose yields measured in the hydrolysate produced for this work (see **Sup. Fig 2** above) or the maximum possible glucose and xylose yields, assuming optimal enzymatic hydrolysis at 20% total solids loading (based on the polysaccharide yields determined by compositional analysis, reported in **Sup. Table 1**).

Equation 3: Percentage of total fermentable sugars fermented

$$\Delta\, \boldsymbol{Sugars_{total}}\, (\%) = \frac{(Glu_i + Xyl_i) - (Glu_f + Xyl_f)}{(Glu_i + Xyl_i)} \times 100$$

Where:

$\Delta Sugars_{total}$ = *Change in sugar concentration after fermentation as a percentage of the total available D-glucose and D-xylose (equivalent to the percentage of total fermentable sugars used). Sugars not fermented by the microorganism are disregarded.*

Glu_i = *Concentration of D-glucose at the start of the fermentation (g/L).*

Xyl_i = *Concentration of D-xylose at the start of the fermentation (g/L).*

Glu_f = *Concentration of D-glucose at the time of maximum product concentration (g/L)*

Xyl_f = *Concentration of D-xylose at the time of maximum product concentration (g/L)*

Equation 4: The product to substrate ratio

$$\boldsymbol{P/S} = \frac{P_{max}}{(Glu_i + Xyl_i) - (Glu_f + Xyl_f)}$$

Where:

P/S = *The Product to Substrate ratio, also known as specific yield*

P_{max} = *The maximum concentration of product (g/L)*

Glu_i = *Concentration of D-glucose at the start of the fermentation (g/L)*

Xyl_i = *Concentration of D-xylose at the start of the fermentation (g/L)*

Glu_f = *Concentration of D-glucose at the time of maximum product concentration (g/L)*

Xyl_f = *Concentration of D-xylose at the time of maximum product concentration (g/L)*

Equation 5: Percentage yield (product yield attained by fermentation, given as a percentage of the theoretical maximum yield from sugars)

$$\boldsymbol{Yield}\ (\%) = \frac{P/S}{Y_{max}} \times 100$$

Where:

% Theoretical Yield = *The product yield as a percentage of the theoretically calculated maximum yield for that fermentation product.*

P/S = *The Product to Substrate ratio (determined from* ***Equation 4****)*

Y_{max} = *The maximum theoretical yield, determined from the literature.*

(Y_{max} ethanol = 0.511; Y_{max} Triacylglycerol = 0.316)

Equation 6: Fermentation productivity

$$\boldsymbol{Productivity\ (g/L.h^{-1})} = \frac{P_{max}}{T_{max}}$$

Where:

Productivity = *The amount of product produced per litre of fermentation medium per hour, also known as process productivity, (g/L.h^{-1})*

P_{max} = *The maximum concentration of product (g/L)*

T_{max} = *Time taken to produce the maximum concentration of product (P_{max}) in h*

Equation 7: Yield per tonne

$$Yield\ (Kg/t) = \boldsymbol{S} \times (\boldsymbol{P}/\boldsymbol{S})$$

Where:

Yield = *The amount of product that could be produced from one tonne of OMSW fibre, based on observed sugar conversion efficiencies, (Kg/t).*

S = *Total substrate (i.e. sugars) available in the feedstock (g/Kg)*

P/S = *The Product to Substrate ratio (g/g) (determined from **Equation 4**)*

References:

1. DEFRA. Digest of Waste and Resource Statistics - 2015 Edition. Department for Environment, Food and Rural Affairs, Department for Food EaRA; 2015 January 2015. Contract No.: PB14292.
2. Sluiter A, Ruiz R, Scarlata C, Sluiter J, Templeton D. Determination of extractives in biomass - laboratory analytical proceedure. National Renewable Energy Laboratories (NREL); 2008. Contract No.: Technical Report NREL/TP-510-42619.
3. Sluiter A, Hames B, Ruiz R, Scarlata C, Sluiter J, Templeton D. Determination of Ash in Biomass. National Renewable Energy Laboratory; 2008. Contract No.: NREL/TP-510-42622.
4. Hames B, Ruiz R, Scarlata C, Sluiter A, Sluiter J, Templeton D. Preparation of Samples for Compositional Analysis. National Renewable Energy Laboratory; 2008. Contract No.: NREL/TP-510-42620.
5. Foster CE, Martin TM, Pauly M. Comprehensive Compositional Analysis of Plant Cell Walls (Lignocellulosic biomass) Part II: Carbohydrates. Journal of Visualized Experiments : JoVE. 2010(37):1837.
6. Fukushima RS, Hatfield RD. Extraction and Isolation of Lignin for Utilization as a Standard to Determine Lignin Concentration Using the Acetyl Bromide Spectrophotometric Method. Journal of Agricultural and Food Chemistry. 2001;49(7):3133-9.
7. Jung S, Rickert DA, Deak NA, Aldin ED, Recknor J, Johnson LA, et al. Comparison of kjeldahl and dumas methods for determining protein contents of soybean products. Journal of the American Oil Chemists' Society. 2003;80(12):1169.
